# Tissue-derived extracellular matrix hydrogels instruct epigenetic adaptation in metastatic colonization

**DOI:** 10.1016/j.bioactmat.2026.08.031

**Published:** 2026-08-26

**Authors:** Heejeong Yoon, Dayoung Kim, Hye-Jin Jeong, Hohyeon Han, Seon-Jin Kim, Jinah Jang, Deok-Beom Jung, Seung-Jae Myung, Chang Sik Yu, In Ho Song, Ju Hae Choi, M. Ryan Corces, Taejoon Kwon, Kyungjae Myung, Jinmyoung Joo, Joo H. Kang, Tae-Eun Park, Seung Woo Cho

**Affiliations:** a Department of Biomedical Engineering, Ulsan National Institute of Science and Technology (UNIST), Ulsan, 44919, Republic of Korea; b Center for Genome Engineering, Institute for Basic Science, Daejeon, 34126, Republic of Korea; c Division of Interdisciplinary Bioscience and Bioengineering, Pohang University of Science and Technology (POSTECH), Pohang, 37673, Republic of Korea; d Department of Mechanical Engineering, Pohang University of Science and Technology, Pohang, 37673, Republic of Korea; e Department of Convergence IT Engineering, Pohang University of Science and Technology, Pohang, 37673, Republic of Korea; f Digestive Diseases Research Center, University of Ulsan College of Medicine, Seoul, 05505, Republic of Korea; g Department of Gastroenterology, Asan Medical Center, University of Ulsan College of Medicine, Seoul, 05505, Republic of Korea; h Division of Colon and Rectal Surgery, Department of Surgery, Asan Medical Center, University of Ulsan College of Medicine, Seoul, 05505, Republic of Korea; i Division of Colorectal Surgery, Department of Surgery, Dongnam Institute of Radiological and Medical Sciences, Busan, 46033, Republic of Korea; j Gladstone Institute of Neurological Disease, San Francisco, CA, 94158, USA; k Gladstone Institute of Data Science and Biotechnology, San Francisco, CA, 94158, USA; l Department of Neurology, University of California San Francisco, San Francisco, CA, 94158, USA; m Center for Genomic Integrity, Institute for Basic Science, Ulsan, 44919, Republic of Korea; n Graduate School of Health Science and Technology, Ulsan National Institute of Science and Technology, Ulsan, 44919, Republic of Korea

## Abstract

The extracellular matrix (ECM) plays a central role in regulating tumor progression and metastatic colonization by providing biochemical and mechanical signals that shape cancer cell fate. However, most organoid culture systems rely on basement membrane extracts that fail to reproduce the tissue-specific extracellular environments encountered during metastasis. Here, we develop tissue-derived decellularized matrix hydrogels to reconstruct organ-specific microenvironments and investigate epigenetic adaptation to ECM cues during metastatic colonization. Patient-derived colorectal cancer organoids cultured in colon-derived matrices exhibited enhanced maintenance of stem-like phenotypes and colon-specific chromatin accessibility landscapes compared with cultures grown in basement membrane extracts, demonstrating improved physiological relevance for primary tumor modeling. When exposed to matrices derived from secondary organs, the organoids showed distinct growth phenotypes accompanied by rapid, tissue-dependent chromatin accessibility remodeling, indicating that ECM composition alone can reshape regulatory programs governing metastatic adaptation. Notably, liver-derived matrices selectively activated hepatocyte nuclear factor 4 alpha (*HNF4A*)–associated transcriptional networks and created a context-specific dependence on c-MET signaling for survival. Functional perturbation of *HNF4A* or c-MET signaling confirmed that both are required for organoid formation specifically within the liver matrix environment. Together, these findings establish tissue-derived matrix hydrogels as instructive bioactive materials that actively regulate cancer cell epigenetic states and reveal microenvironment-specific therapeutic vulnerabilities during early metastatic colonization.

## Highlights

• Tissue-derived matrix hydrogels reconstruct organ-specific tumor microenvironments.
• Matrix composition instructs epigenetic remodeling during metastatic adaptation.
• Colon-derived matrices preserve colorectal cancer–specific chromatin states.
• Liver-derived matrices activate HNF4A-dependent regulatory programs.
• Liver matrix conditions create a context-specific vulnerability to c-MET signaling.

## Graphical abstract

**Figure:**
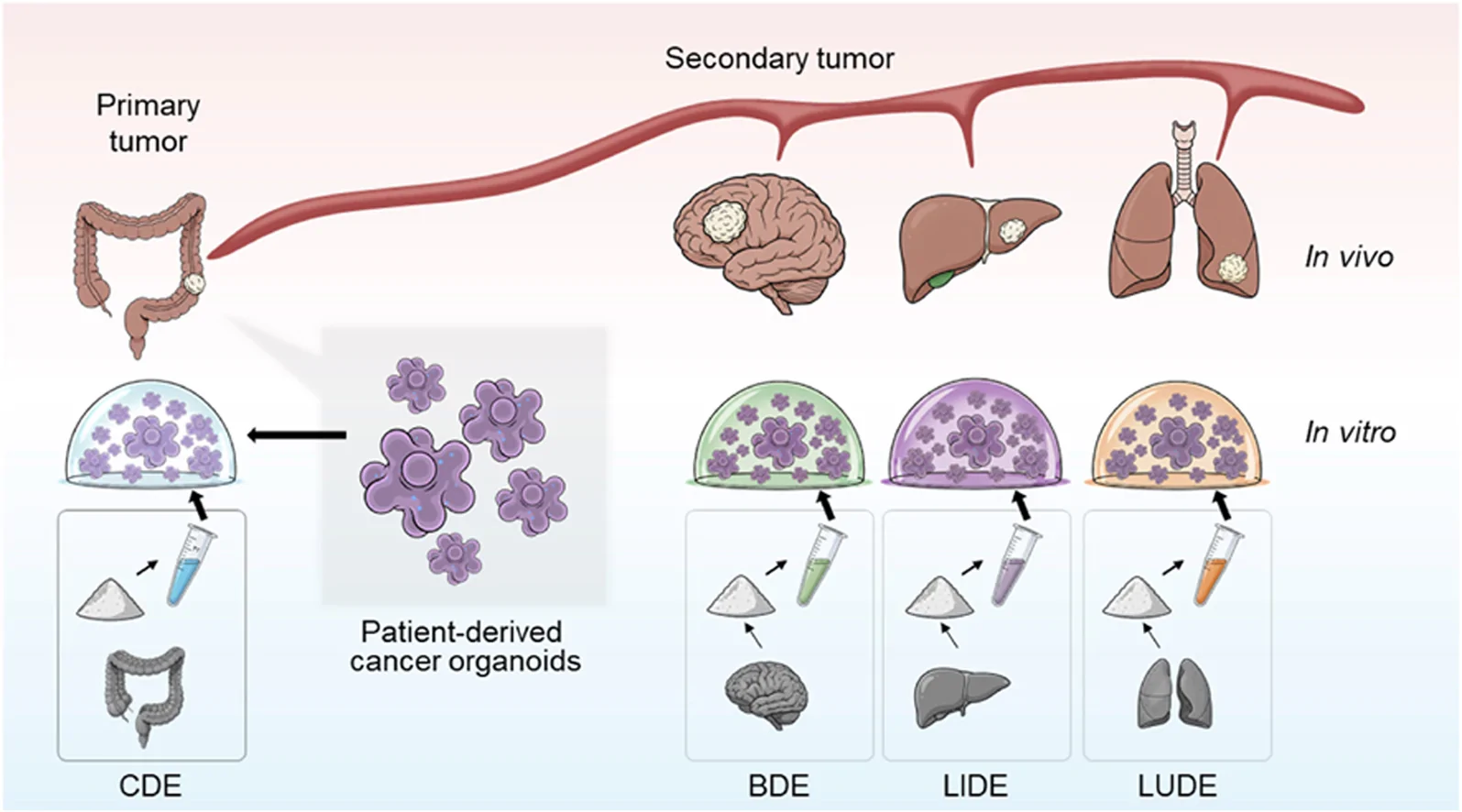

## 1 Introduction

Metastasis remains the leading cause of cancer-related mortality, yet effective strategies to control this process are still limited [1]. A major bottleneck in the metastatic cascade is metastatic colonization, during which disseminated tumor cells must adapt to and survive within a foreign tissue microenvironment [2]. This step is highly inefficient, with only a small fraction of disseminated cells successfully establishing secondary tumors. Increasing evidence suggests that successful colonization critically depends on interactions between tumor cells and the surrounding microenvironment, particularly the extracellular matrix (ECM), which shapes tumor cell survival, plasticity, and long-term outgrowth [3,4].

Beyond its structural role, the ECM functions as a dynamic signaling platform that regulates tumor progression through biochemical and biomechanical cues [5,6]. ECM stiffness has been shown to modulate cancer cell behavior through mechanotransduction pathways such as YAP/TAZ signaling, while ECM composition further influences metastatic fate [7,8]. Fibronectin- and collagen I–rich matrices promote integrin-mediated signaling and stem-like phenotypes, whereas laminin- and hyaluronan-enriched environments are associated with tumor dormancy and therapeutic resistance [[9], [10], [11]]. In addition, the ECM actively remodels the metastatic niche by regulating epithelial-mesenchymal transition/mesenchymal-epithelial programs [12] and by storing and releasing growth factors such as TGF-β [13], VEGF [14], and FGF [15]. Despite these advances, how tissue-specific ECM cues at secondary sites drive tumor cell adaptation at the molecular level remains poorly understood.

Organoids are three-dimensional *in vitro* models derived from patient tissues or stem cells that precisely recapitulate the structural complexity and physiological functions of native organs [[4], [5], [6]], enabling their broad application in studies of tissue-specific physiological processes, including epithelial barrier function, drug delivery, and disease modeling [[16], [17], [18], [19]]. Patient-derived organoids (PDOs), in particular, have emerged as a powerful platform for modeling cancer biology, as they preserve tumor architecture, genetic heterogeneity, and therapeutic responses more faithfully than conventional cell lines [20]. However, most three-dimensional PDO cultures rely on basement membrane–derived matrices such as Matrigel™ or Geltrex™, which are enriched in laminin and collagen IV and lack the compositional and mechanical diversity of native tissues [21]. As a result, these systems inadequately capture the tissue-specific ECM landscapes encountered during metastasis. To address this limitation, decellularized extracellular matrix (dECM), which retains the biochemical composition of tissue-specific ECM, has been proposed as a more physiologically relevant alternative [22,23]. Previous studies have demonstrated that dECM-based cultures correlate with organ-specific metastatic tendencies and drug responses, suggesting that ECM composition can functionally influence metastatic adaptation. However, the mechanisms by which tissue-specific ECM cues are transduced into stable regulatory programs remain largely unresolved.

Metastatic colonization is a highly dynamic process that requires rapid transcriptional adaptation governed at the chromatin level [24,25]. Changes in chromatin accessibility often precede and dictate transcriptional responses, thereby positioning epigenetic regulation as a central driver of cellular plasticity [26,27]. Accordingly, metastatic colonization is increasingly viewed as an epigenetically regulated process. During metastatic progression, cancer cells undergo extensive remodeling of regulatory landscapes to acquire and maintain colonization capacity across diverse cancer types [28]. For instance, temporal shifts in chromatin accessibility defined the transcriptional states required for metastatic colonization [25] and transcription factor mediated activation of distal regulatory elements promotes metastatic competence [24,29].

Importantly, accumulating evidence indicates that ECM-responsive signaling is tightly coupled to epigenetic regulation. ECM-responsive pathways including YAP/TAZ have been shown to interact directly with chromatin-remodeling machinery [30] and enhancer elements [31]. In addition, both ECM stiffness and composition have been implicated in modulating chromatin accessibility, suggesting a direct link between extracellular cues and epigenetic remodeling [[32], [33], [34]].

**Fig. 1:**
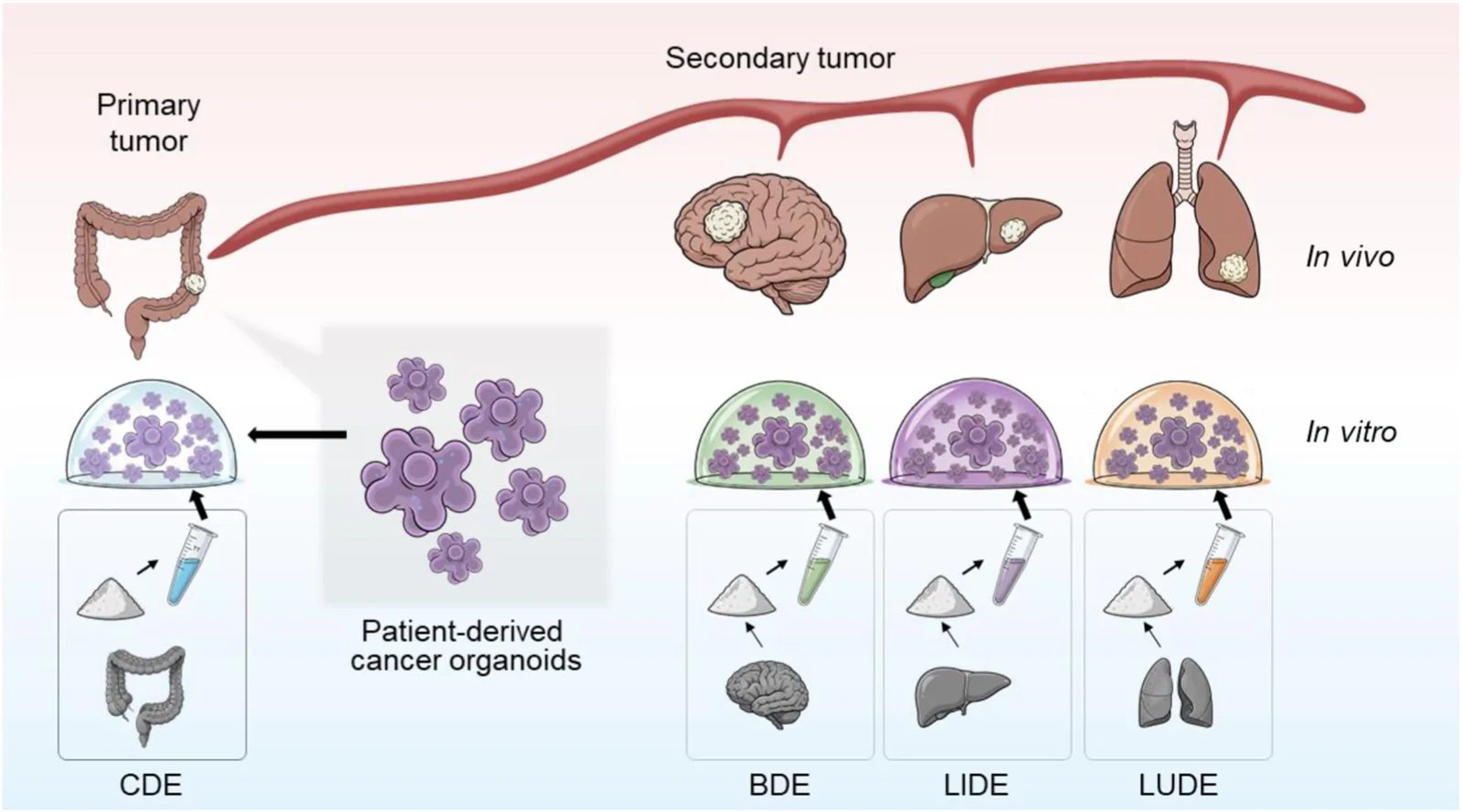
**Schematic illustration of the experimental strategy used to investigate organ-specific extracellular matrix (ECM) influences on early metastatic colonization.** Patient-derived colorectal cancer organoids (PDCOs) were cultured in decellularized ECM (dECM) hydrogels derived from colon (CDE), brain (BDE), liver (LIDE), and lung (LUDE) tissues. This platform recapitulates tissue-specific extracellular microenvironments encountered during early metastatic seeding. By comparing tumor cell behavior across organ-specific dECMs, we evaluated how differential ECM environments contribute to metastatic adaptation within distinct secondary organ niches. The schematic was created using FigureLabs (http://www.figurelabs.ai/) and further adapted by the authors.

Although ECM is recognized as a critical determinant of metastatic colonization, its role in metastatic adaptation has primarily been characterized at the phenotypic level. Consequently, a substantial gap remains in understanding the role of organ-specific ECM microenvironments in regulating the epigenetic plasticity of cancer cells to enable metastasis. We therefore hypothesized that the biochemical composition of tissue-specific ECM is sufficient to instruct chromatin accessibility remodeling in disseminated tumor cells, thereby driving the organ-specific epigenetic programs that underlie metastatic adaptation. To test this hypothesis, we investigated how tissue-specific ECM at primary and secondary sites shape the epigenetic landscape of cancer cells during metastatic colonization (Fig. 1). We reconstructed tumor and metastatic microenvironments using decellularized ECM derived from the colon, liver, lung, and brain, and cultured patient-derived colorectal cancer organoids (PDCOs) within these matrices. To resolve adaptation at the regulatory level, we performed Assay for Transposase-Accessible Chromatin using sequencing (ATAC-seq) [35], in which a hyperactive Tn5 transposase preferentially inserts sequencing adapters into nucleosome-free DNA, generating genome-wide maps of accessible chromatin including promoters and enhancers. As chromatin accessibility reflects the potential regulatory activity of transcription factor binding sites, it provides a sensitive readout of the regulatory remodeling that precedes and accompanies transcriptional adaptation [36,37]. This approach enabled us to identify organ-specific epigenetic programs and uncover microenvironment-dependent regulatory vulnerabilities associated with metastatic adaptation. Together, these analyses establish the organoid–dECM platform as a physiologically relevant system that more faithfully recapitulates organ-specific microenvironments for modeling tumor adaptation during metastatic colonization.

## 2 Results and discussion

### 2.1 Organ-specific dECM preserves native ECM signatures

**Fig. 2:**
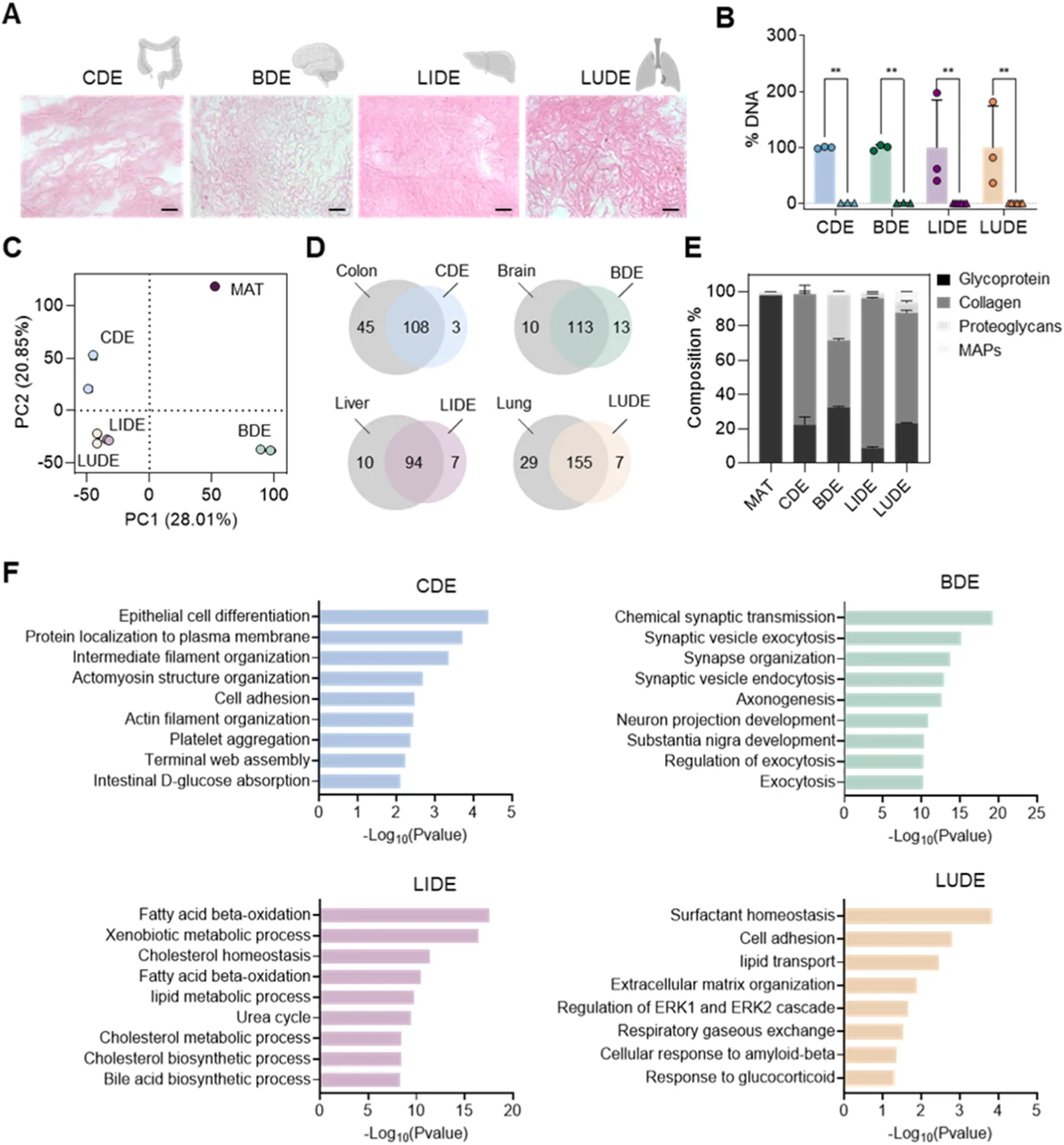
**Characterization of decellularized ECM (dECM).** (A) Representative H&E images of dECMs. Scale bar, 50 μm. (B) DNA content in tissues before and after decellularization. (C-G) Proteomic analysis of native tissues, dECMs, and Matrigel (MAT). Data are presented as mean ± standard deviation (SD) (n ≥ 3). Statistical significance was determined by two-way ANOVA followed by Bonferroni's post-hoc test for multiple comparisons. **p < 0.01. (C) Principal component analysis of dECM hydrogels (CDE: colon-derived dECM, BDE: brain-derived dECM, LIDE: liver-derived dECM, and LUDE: lung-derived dECM) and MAT. (D) Venn diagrams showing the total number of Matrisome proteins identified in native tissues and corresponding dECM. Protein retention rates relative to the corresponding native tissues were 70.6% (CDE), 91.9% (BDE), 90.4% (LIDE), and 84.2% (LUDE). (E) Relative composition of Matrisome protein subcategories in each dECM. MAP, Matrisome-associated proteins. (F) Gene Ontology analysis of proteins overlapping between those identified in proteomic analysis and tissue-enriched protein signatures. Data represent normalized proteomic analyses from n = 2 independent biological replicates.

The dECM hydrogels were prepared from porcine colon [38], brain [39], liver [40], and lung [41] tissues using a slightly modified version of previously established protocols, serving as organ-specific ECM scaffolds. Porcine tissues were selected because they provide an abundant, scalable, and translationally validated source for fabricating organ-specific dECM scaffolds [42,43]. The general procedure for dECM hydrogel preparation involved tissue mincing, removal of cellular components, freeze-drying, pepsin digestion, neutralization, and thermo-gelation. While these core steps were consistent across all tissues, specific protocols were adjusted based on the unique characteristics of each tissue type. Absence of nuclei in H&E-stained sections of each dECM further confirmed successful removal of cellular components (Fig. 2A). Quantification of double-stranded DNA (dsDNA) confirmed effective decellularization, showing significantly lower dsDNA content in dECM samples compared to their native counterparts (Fig. 2B). Additional biochemical characterization of the dECM hydrogels was performed by assessing glycosaminoglycan (GAG) content and glycosylation patterns. GAG content was reduced in all tissue-derived dECM compared with native tissues (Supplementary Fig. 1A). Lectin blot analysis further revealed distinct glycosylation profiles among the different tissue-derived dECMs, demonstrating tissue-dependent differences in glycosylation patterns that were also distinct from those of MAT (Supplementary Fig. 1B).

Proteomic analysis (LC-MS/MS) of dECM revealed tissue-specific protein profiles (Fig. 2C–F and Supplementary Fig. 1C–F). Principal component analysis revealed clear separation between the different dECM samples and Matrigel (MAT), indicating distinct ECM compositions for each source tissue (Fig. 2C). A substantial number of Matrisome [44] proteins were shared between the decellularized ECMs and their corresponding native tissues, with 108, 113, 94, and 155 proteins commonly identified in colon- (CDE), brain- (BDE), liver- (LIDE), and lung-derived ECMs (LUDE), respectively (Fig. 2D). Moreover, Matrisome proteins were substantially preserved across all decellularized tissues, with protein retention rates of 70.6%, 91.9%, 90.4%, and 84.2% in the CDE, BDE, LIDE, and LUDE, respectively, relative to their corresponding native tissues. Protein profiling of Matrisome components revealed that compared to MAT—composed mostly of laminin and glycoproteins (∼98%)—tissue-derived dECMs preserved a broader range of Matrisome proteins, including collagens, glycoproteins, and proteoglycans (Fig. 2E). These results confirm a high degree of similarity in the composition of Matrisome proteins between each dECM and its native tissue. Each dECM also overlapped with tissue-enriched proteins from the Human Protein Atlas [45], highlighting the tissue specificity of each dECM and suggesting that porcine-derived dECMs retain ECM components associated with human tissue-specific characteristics (Supplementary Fig. 1C). Compared to MAT—which contained less than 10% tissue-specific proteins—dECMs retained a significantly higher proportion of native, tissue-enriched proteins. These overlaps support the preservation of key tissue-specific ECM features during the decellularization process.

The compositional analysis of Matrisome categories further highlighted differences across dECM types (Fig. 2E). For instance, LUDE and BDE were enriched in ECM glycoproteins and proteoglycans, aligning with the known structural flexibility and dynamic signaling. In contrast, CDE and LIDE exhibited a higher proportion of core Matrisome proteins such as collagens, consistent with more dense and fibrous architecture of these tissues. Normalized abundance plots of the top 10 most abundant Matrisome proteins in each dECM and MAT further supported the tissue specificity of the dECMs (Supplementary Fig. 1D and E). For example, CDE was enriched in collagen subtypes such as COL1 and COL3-6, as well as FBN. In contrast, BDE prominently featured proteoglycans like HSPG2, which are known to be abundant in the brain ECM [39,46]. LIDE showed high abundance of collagen subtypes along with DPT, a regulator of collagen fibrillogenesis in liver [47]. LUDE contained abundant collagens and EMILIN1, a protein highly expressed in elastin-rich tissues like lung. Heatmap analysis of the top 20 detected proteins within the glycoprotein, collagen, and proteoglycan subcategories further confirmed distinct tissue-specific Matrisome signatures across the dECMs, with minimal batch-to-batch variation and clear distinction from MAT (Supplementary Fig. 1F).

Gene Ontology biological process analysis (Fig. 2F) further revealed distinct biological functions associated with each dECM. For example, CDE was enriched in processes related to epithelial cell differentiation, cytoskeleton organization, and intermediate filament organization. BDE supported pathways related to nervous system and neuron projection development. LIDE was linked to lipid metabolism, fatty acid β-oxidation, and cellular oxidant detoxification. LUDE was associated with surfactant homeostasis and respiratory gas exchange. Together, these findings demonstrate that tissue-specific porcine-derived dECMs preserve native ECM composition and biological functions, consistent with previous studies [48], supporting their use as PDCO scaffolds for modeling tissue-specific ECM environments. Nevertheless, because the current platform is based on porcine-derived dECMs, residual species-specific ECM components, including glycosylation patterns or matrix-associated molecules, may still influence human cell responses. Therefore, further validation using human tissue-derived matrices will be important to establish the translational relevance of this platform.

The physical properties of the tissue-specific dECM hydrogels were characterized by rheological measurements, stress relaxation analysis, SEM imaging, swelling behavior, dry mass, and dextran release assays (Supplementary Fig. 2). In all tissue-specific dECM hydrogels, the storage modulus (G′) consistently exceeded the loss modulus (G″) across the tested strain and frequency ranges, indicating predominantly elastic and stable gel-like behavior. All tissue-specific dECM hydrogels displayed low viscoelastic moduli within a similar physiological range (approximately 50–150 Pa), consistent with soft tissue microenvironments. Stress relaxation analysis demonstrated comparable relaxation behaviors among the dECM hydrogels, indicating similar time-dependent viscoelastic responses. Representative SEM images revealed comparable fibrous microstructures among the tissue-specific dECM hydrogels. Swelling and dry mass analyses showed no marked differences among the dECM hydrogels, although CDE exhibited a slight tendency toward increased swelling. Similarly, dextran release profiles were largely comparable among the dECM hydrogels, with CDE exhibiting a slightly higher release profile compared with the other dECM hydrogels; however, the overall differences were limited. Collectively, these results indicate that tissue-specific dECM hydrogels maintain comparable physical properties despite distinct ECM compositions, providing a consistent hydrogel framework for studying cellular responses to tissue-specific ECM cues.

### 2.2 Colon-derived ECM supports colorectal cancer-specific phenotypes

**Fig. 3:**
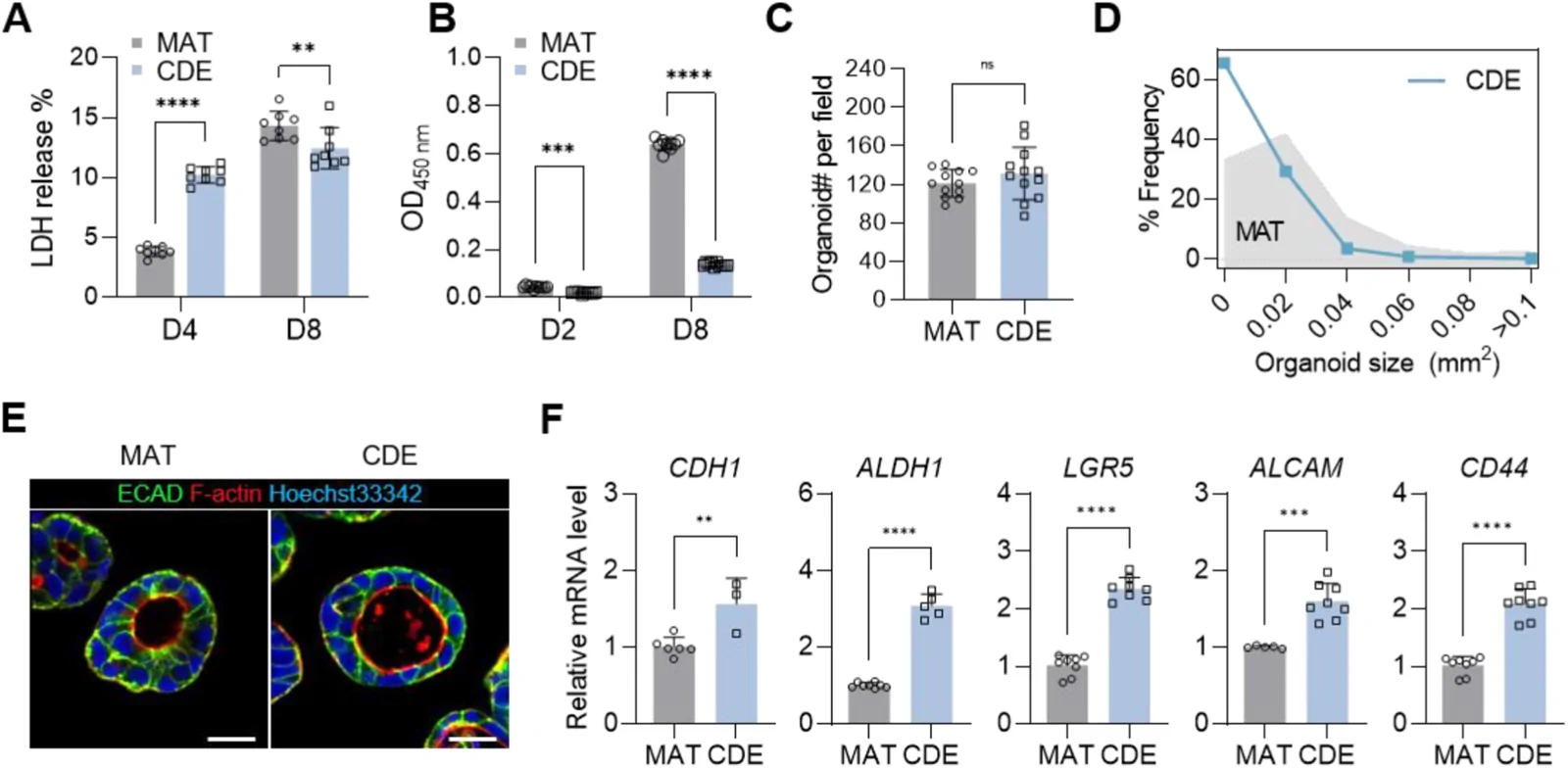
**Growth and phenotypic characterization of PDCOs cultured in MAT and CDE hydrogels.** (A) Lactate dehydrogenase (LDH) release assay of PDCOs cultured in CDE and MAT on days 4 and 8. (B) Cell proliferation and metabolic activity of PDCOs cultured in CDE and MAT on days 2 and 8, assessed using the WST-8 assay. The organoid formation efficiency (C) and size distribution (D) of PDCOs cultured in MAT and CDE. (E) Representative immunofluorescence images of PDCOs cultured in MAT and CDE on day 8, stained for E-cadherin (ECAD, green), F-actin (red), and Hoeschst 33342 (blue). Scale bars, 20 μm. (F) Relative gene expression of PDCOs cultured in MAT and CDE on day 8, normalized to MAT. Data are presented as mean ± SD (n ≥ 3). Statistical significance was determined by Student's t-test for two-group comparisons, or two-way ANOVA followed by Bonferroni's post hoc test for multiple comparisons. **p < 0.01; ***p < 0.001; ****p < 0.0001.

Before investigating the epigenetic adaptation of PDCOs within secondary tissue-derived dECM scaffolds, we first assessed whether these scaffolds provide a tissue-specific microenvironment by comparing the cellular behavior and epigenetic profiles of PDCOs cultured in CDE as a native ECM versus MAT. PDCOs cultured in CDE initially exhibited significantly elevated lactate dehydrogenase (LDH) release on day 4 compared to those in MAT, indicating an early cellular stress response to the complex ECM environment (Fig. 3A). By day 8, LDH levels were significantly lower than that in MAT, suggesting that the cells had adapted to the native ECM cues. WST-8 analysis in CDE also showed a steady increase in metabolic activity from day 2 through day 8, reflecting progressive cell adaptation and growth within the tissue-specific microenvironment (Fig. 3B). This early selection pressure likely allowed only the cells capable of adjusting to the tissue-specific microenvironment to survive and form organoids. In contrast, PDCOs cultured in MAT—an ECM rich in laminin (Supplementary Fig. 1B), a key component for epithelial organoid development through integrin-mediated interactions involving RGD motifs [[49], [50], [51]]—exhibited more rapid growth at early stages.

Importantly, PDCOs formed in the CDE exhibited a higher proportion of Ki-67–positive cells at day 8 compared with those cultured in MAT (Supplementary Fig. 3A), indicating that the surviving cell population not only adapted to the native ECM scaffold but also maintained robust proliferative activity. In addition, a comparable number of organoids formed under both conditions (Fig. 3C); however, PDCOs cultured in CDE displayed a size distribution with a predominance of smaller organoids compared with those grown in MAT (Fig. 3D). Despite these differences in growth characteristics, PDCOs in both conditions maintained well-developed epithelial structures with intact tight junction, as demonstrated by E-cadherin (Fig. 3E) and ZO-1 staining (Supplementary Fig. 3B). Collectively, these findings indicate that the CDE hydrogel supports PDCO survival and epithelial organization without inducing apoptosis, validating its stability as a supportive culture environment.

Building on this, we next examined whether the CDE microenvironment also supports the maintenance of cancer stem-like cells, which are critical for preserving heterogeneity in colorectal cancer (CRC) [52]. To assess stemness, we performed quantitative polymerase chain reaction (qPCR) to compare the expression levels of key CRC stem-like cell markers, including leucine-rich repeat containing G protein-coupled receptor 5 (*LGR5*) [53], aldehyde dehydrogenase (*ALDH1*) [54], activated leukocyte cell adhesion molecule (*ALCAM*) [55], and CD44 [56] (Fig. 3F). The results showed a notable upregulation of these markers in PDCOs cultured in CDE, indicating the enhanced capacity of CDE to preserve stem-like cell populations and maintain the intrinsic properties of PDCOs [57]. Immunofluorescence staining of representative stem-like cell markers, LGR5 and ALDH1, further visualized the expression patterns of these markers in PDCOs cultured under CDE and MAT conditions (Supplementary Fig. 3C). These findings demonstrate that the CDE hydrogel more faithfully recapitulates the native colonic microenvironment than MAT, thereby more effectively supporting cancer stem-like cell maintenance and tumor heterogeneity in PDCOs, highlighting its potential as a physiologically relevant platform for CRC modeling.

### 2.3 Colon-derived ECM supports CRC-specific epigenetic fidelity

While the results demonstrated phenotypic adaptations of PDCOs to the CDE microenvironment, including enhanced epithelial characteristics and stem cell marker expression, the underlying mechanisms remained unclear. To elucidate how CDE drives these changes at the molecular level, we performed chromatin accessibility profiling using ATAC-seq to assess ECM-induced epigenetic reprogramming.

**Fig. 4:**
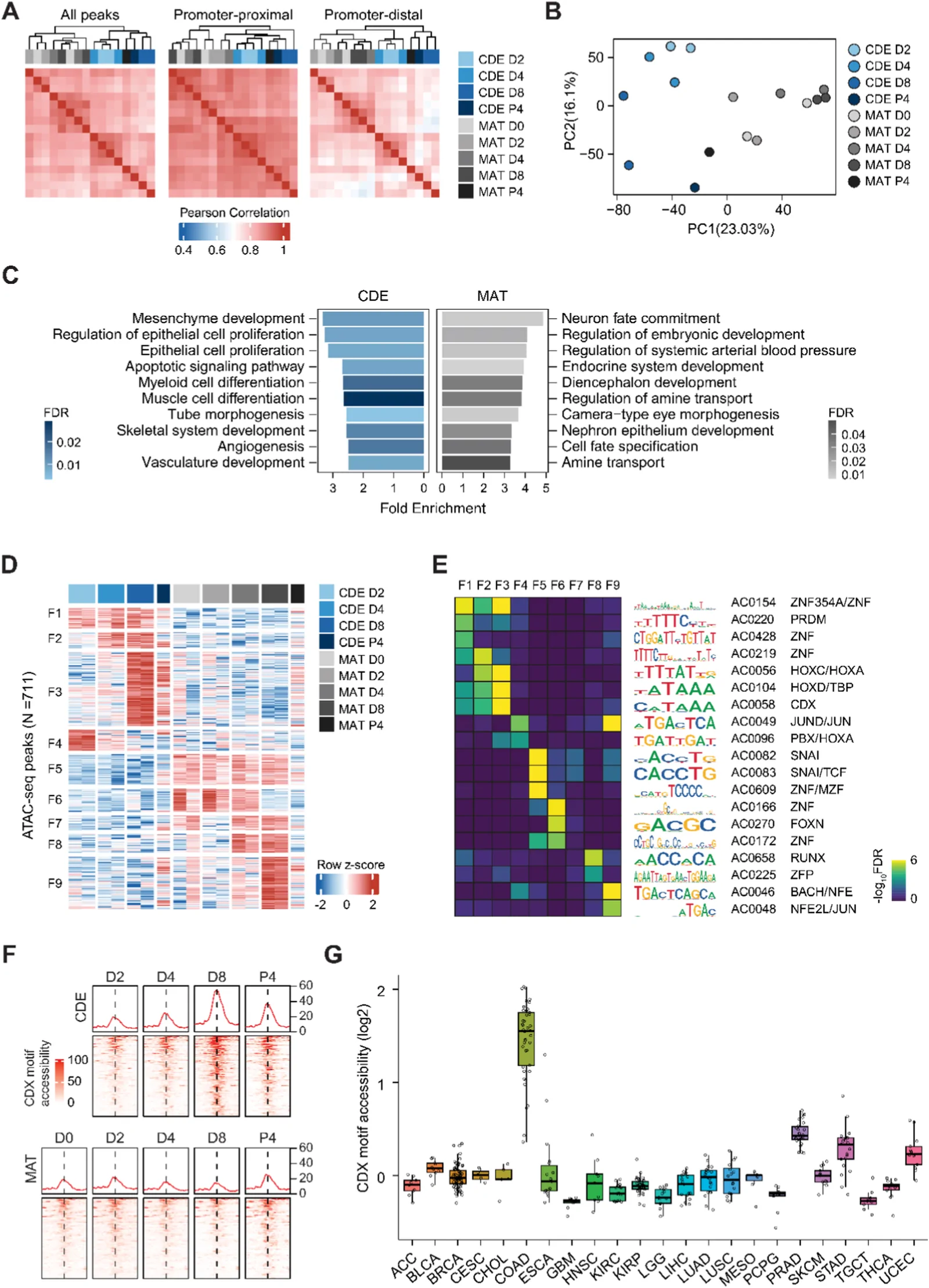
**Colon-derived dECM supports CRC-specific epigenetic signatures.** (A) Pearson correlation analysis of chromatin accessibility profiles across PDCOs cultured in CDE and MAT, harvested at multiple timepoints. Analysis includes all peaks (n = 73,118), promoter regions (defined as −1 kb to +100 bp from transcription start sites, n = 22,831), and distal regulatory elements (n = 50,287). Hierarchical clustering (top) demonstrates distinct separation between MAT and CDE culture conditions. (B) Principal component analysis of regulatory elements distinguishing CDE and MAT conditions across multiple harvest time points. (C) Gene Ontology enrichment analysis of biological processes associated with differentially accessible regions in CDE and MAT conditions. The top 10 enriched terms are ranked by fold enrichment, with color intensity representing false discovery rate (FDR). (D) Heatmap visualization of differential chromatin accessibility analysis using feature binarization, revealing 711 peaks with either unique accessibility patterns or gradual increases in chromatin openness over time. Distinct feature categories are labeled on the left. (E) Transcription factor (TF) motif enrichment was performed separately within each feature group defined in Fig. 4D, in order to distinguish transcription factors associated with transient, early accessibility gains from those linked to sustained or progressively increasing accessibility during adaptation. The heatmap displays -log10 transformed, Benjamini-Hochberg adjusted p-values for enriched motifs. Representative TF binding motifs (middle panel) are shown alongside their corresponding transcription factors (right panel). (F) Aggregate plot of *CDX* motif accessibility across different groups. Upper panel: Average chromatin accessibility profile centered on *CDX* motifs present in F1, F2 and F3 feature peaks. Lower panel: Heatmap showing mean accessibility scores across corresponding samples, with the *CDX* motif center indicated by the dotted line and ±1 kb flanking regions. (G) Boxplot of average chromatin accessibility of *CDX* motifs in F1, F2, and F3 feature peaks across 23 cancer types in TCGA [58]. Each dot represents a patient belonging to a specific cancer type.

We collected samples at multiple time points to capture the temporal dynamics of adaptation. Day 0 samples represented PDCOs maintained in MAT, while subsequent samples were harvested on days 2, 4, and 8 following transfers to CDE or continued culture in MAT. Two biological replicates were analyzed for each condition and time point to ensure robust comparison. Hierarchical clustering of ATAC-seq data revealed distinct epigenetic landscapes between growth conditions, with clear separation between CDE and MAT cultures across all accessible chromatin regions (Fig. 4A). This separation was most pronounced in promoter-distal regulatory elements, indicating substantial reprogramming of gene regulatory networks under CDE conditions.

CDE induced time-dependent shifts in chromatin accessibility (Fig. 4B), leading to the identification of differentially accessible regions (DARs) between CDE and MAT cultured PDCOs (Supplementary Fig. 4A and B). Notably, DARs enriched in CDE cultures exhibited increased accessibility specifically in colon adenocarcinomas compared to other cancer types from the TCGA datasets [58] (Supplementary Fig. 4C), while MAT-activated DARs showed broad accessibility across various cancer types (Supplementary Fig. 4D). These findings suggest that CDE-induced chromatin alterations specifically recapitulate colon cancer and normal colon tissue regulatory features rather than generic cancer-associated changes. Gene Ontology analysis further supported this tissue-specific reprogramming, revealing enrichment of colon epithelial regulatory programs in CDE cultures, whereas MAT cultures retained more generic stem-like regulatory signatures. (Fig. 4C).

To identify the potential transcription factors (TFs) regulating these epigenetic changes, we performed TF motif enrichment analysis on the DARs. While both conditions showed diverse TF motif enrichment patterns over time (Fig. 4D–E), their regulatory signatures were distinctly different. In MAT cultures, we consistently observed a sustained enrichment of motifs associated with undifferentiated states and cellular plasticity. SNAI family members—well-characterized master regulators of the epithelial-mesenchymal transition and the maintenance of stem-like features [59,60]—exhibited high enrichment scores (Fig. 4E).

In contrast, CDE cultures demonstrated a progressive enrichment of tissue-specific TFs, including CDX1 (CDX; Fig. 4E), a known regulator of colon development and epithelial homeostasis [61]. The accessibility of CDX1 motifs in CDE cultures increased over time (Fig. 4F), consistent with epigenetic adaptation to the tissue-specific dECM environment. Furthermore, the CDX1 motifs that gained accessibility in CDE cultures were also specifically accessible in colon cancer tissues (Fig. 4G). To determine whether these accessibility changes occur at functional regulatory elements, we profiled the active enhancer mark H3K27ac by Cleavage Under Targets and Tagmentation (CUT&Tag) in MAT (day 0 and day 8) and CDE (day 8). Genome-wide chromatin accessibility was positively correlated with H3K27ac enrichment (Supplementary Fig. 4E), and CDX1 motif-containing regions that gained accessibility in CDE exhibited significantly higher H3K27ac levels than in MAT (1.31-fold over MAT; Supplementary Fig. 4F), providing independent evidence that the accessibility changes identified by ATAC-seq occur at transcriptionally active regulatory elements.

Collectively, these results demonstrate that CDE establishes a tissue-specific microenvironment through biochemical cues that systematically reshape the epigenetic landscape of colon cancer cells. This epigenetic reprogramming promotes epithelial characteristics while preserving cancer stem cell properties, providing mechanistic insight into how tissue-specific ECM cues shape lineage-specific regulatory programs and cellular identity.

### 2.4 PDCO behavior in secondary tissue-derived ECM hydrogels

**Fig. 5:**
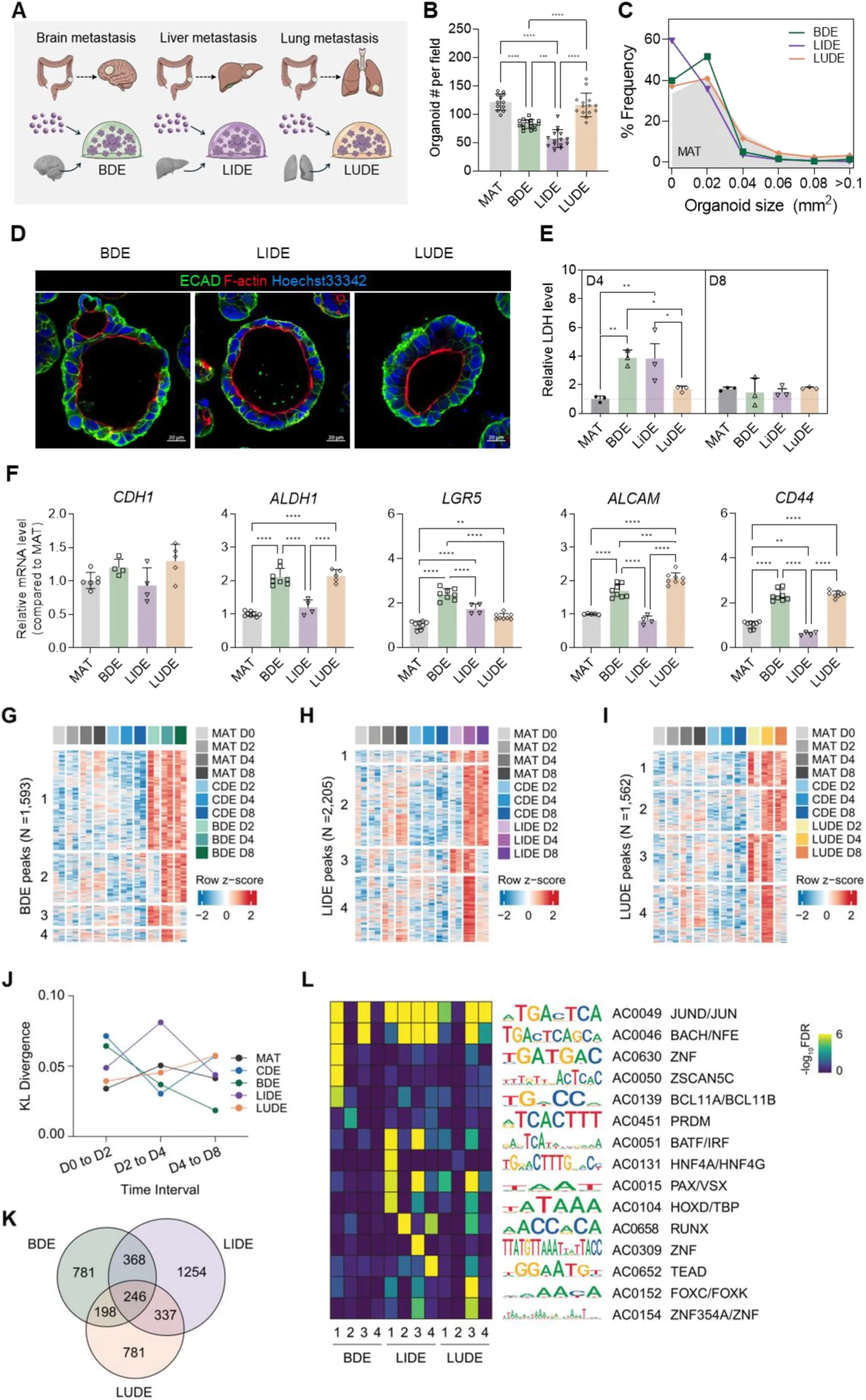
**PDCOs cultured in tissue-specific dECMs modeling secondary metastatic niches display unique chromatin accessibility signatures.** (A) Schematic illustration of PDCOs cultured in BDE, LIDE, and LUDE dECM hydrogels to recapitulate organ-specific metastatic microenvironments *in vitro*. (B) Organoid formation efficiency and (C) size distribution of PDCOs cultured in tissue-specific dECMs. (D) Representative immunofluorescence images of PDCOs cultured in BDE, LIDE, and LUDE on day 8, stained for E-cadherin (ECAD, green), with F-actin (red) and Hoeschst33342 (blue). Scale bars, 20 μm. (E) Lactate dehydrogenase (LDH) release from PDCOs cultured in each tissue-specific dECM. (F) Relative expression of stemness-associated genes in PDCOs on day 8, normalized to MAT controls. Data are presented as mean ± SD (n ≥ 3). Statistical significance was determined by one-way ANOVA followed by Tukey's post hoc test for multiple comparisons. *p < 0.05; **p < 0.01; ***p < 0.001; ****p < 0.0001. (G-I) Heatmaps of differentially accessibility peaks that are either uniquely accessible or show gradual opening across time in (G) BDE, (H) LIDE, and (I) LUDE conditions. Peak score is scaled by row Z-score. Sample groups and time points (days 0, 2, 4, and 8) are indicated above each heatmap and include MAT, CDE, and the respective tissue-specific dECM. (J) Kullback-Leibler divergence quantifying changes in chromatin accessibility distributions across consecutive time intervals (D0–D2, D2–D4, and D4–D8) for each culture condition. (K) Venn diagram showing the overlap of differentially accessible regions (DARs) among BDE, LIDE, and LUDE in Fig. 5G–I. (L) TF motif enrichment analysis of condition-specific DARs. The heatmap shows -log10(FDR) values (Benjamini-Hochberg adjusted) for enriched motifs across peak groups (1–4) in each dECM condition, corresponding to the temporal categories in Fig. 5G–I. Representative TF-binding motifs (middle) and their corresponding TF (right) are shown.

Next, we examined how PDCOs adapt to ECM environments representative of potential secondary metastatic sites, including the liver and lung as major metastatic sites in colorectal cancer and the brain as a less frequent but clinically significant metastatic site associated with poor prognosis [62,63]. As a first step prior to comparative ATAC-seq analysis, we assessed the phenotypic characteristics of PDCOs cultured in BDE, LIDE, and LUDE hydrogels (Fig. 5A).

Organoid formation varied across ECM types (Fig. 5B). The number of organoids was lowest in LIDE, followed by BDE, while LUDE supported organoid formation at levels comparable to MAT. Correspondingly, a higher proportion of small-sized organoids (<0.02 mm^2^) was observed in LIDE and BDE compared to LUDE (Fig. 5C), possibly due to the lower levels of RGD-containing ECM components in LIDE and BDE, whereas LUDE is enriched in EMILIN1 and TNC, as revealed by ECM proteomic analysis (Supplementary Fig. 1C).

Across all ECM conditions, PDCOs formed epithelial structures with intact tight junctions, as confirmed by E-cadherin (Fig. 5D) and ZO-1 staining (Supplementary Fig. 5). Consistent with these observations, no significant differences in CDH1 mRNA expression were detected among the groups, indicating that PDCOs maintain robust epithelial organization irrespective of ECM composition.

LDH release was elevated in BDE and LIDE on day 4 (Fig. 5E), reflecting an early stress response; however, LDH levels returned to baseline by day 8, suggesting that these ECMs do not induce persistent cytotoxicity and are permissive for organoid viability over time. Collectively, these observations suggest that early ECM–cell interactions are an important factor associated with tumor cell adaptation to new microenvironments.

The stem cell marker expression varied across ECM conditions. PDCOs cultured in BDE and LUDE exhibited higher expression levels of *LGR5*, *ALDH1*, and *ALCAM* compared to those in MAT, indicating that these ECMs—similar to CDE—may better support the maintenance of stem-like phenotypes (Fig. 5F). Interestingly, PDCOs cultured in LIDE deviated from this pattern, displaying elevated *LGR5* expression, in contrast to the other markers, which remained unchanged or were downregulated.

Collectively, these findings demonstrate that PDCOs exhibit distinct responses when cultured in different dECMs, as evidenced by marked differences in organoid formation, viability, and stemness-associated marker expression. Such variability likely reflects not only the biochemical heterogeneity of tissue-specific ECMs but also differing levels of permissiveness or constraint imposed by native microenvironments on disseminated tumor cells. While clinical observations indicate that the liver is the most common site of CRC metastasis, our results suggest that BDE and LIDE are not uniformly supportive of early tumor outgrowth. The behavior of PDCOs within these matrices may therefore model an initial phase of resistance to metastatic colonization, implying that adaptive cellular reprogramming is required for CRC cells to survive and establish in secondary tissues. These observations raised the possibility that successful adaptation to secondary ECM environments may require extensive epigenetic reprogramming.

### 2.5 Secondary tissue-derived ECM induces epigenetic reprogramming of PDCOs

Given that chromatin accessibility remodeling is crucial for cellular adaptation to a novel microenvironment during metastatic colonization [24,64,65], we next asked how PDCOs remodel their epigenome when cultured in tissue-specific dECM environments. Compared with MAT controls and CDE, ATAC-seq revealed rapid and extensive chromatin accessibility reorganization across BDE, LIDE, and LUDE (Fig. 5G–I and Supplementary Fig. 6A–B). Each dECM hydrogel induced a distinct chromatin landscape relative to MAT and CDE (Supplementary Fig. 6C–E), indicating that ECM composition drives divergent accessibility programs.

The temporal dynamics of chromatin accessibility remodeling differed across ECM types (Fig. 5J). Differential accessibility analysis further supported the tissue specificity of these responses. Across dECM types, CDE, and MAT, overlap between DAR group was limited: only 246 DARs were shared across all conditions, representing a small fraction of each tissue-associated DAR set (lung 15.7%, brain 15.4%, liver 11.2%) (Fig. 5K). TF motif enrichment analysis of these tissue-specific DARs identified candidate regulators associated with these distinct epigenetic states (Fig. 5L). Motifs for JUND/JUN and BACH/NFE were enriched across multiple environments, whereas tissue-preferential enrichment was observed for *ZNF* motifs in BDE, BATF/IRF and HNF4A/HNF4G in LIDE, and FOXC/FOXK in LUDE. These findings demonstrate that PDCOs engage tissue-specific chromatin accessibility programs when cultured in distinct dECMs, consistent with ECM-driven epigenetic adaptation.

### *2.6* *HNF4A* mediates PDCO adaptation within liver-derived ECM environments

**Fig. 6:**
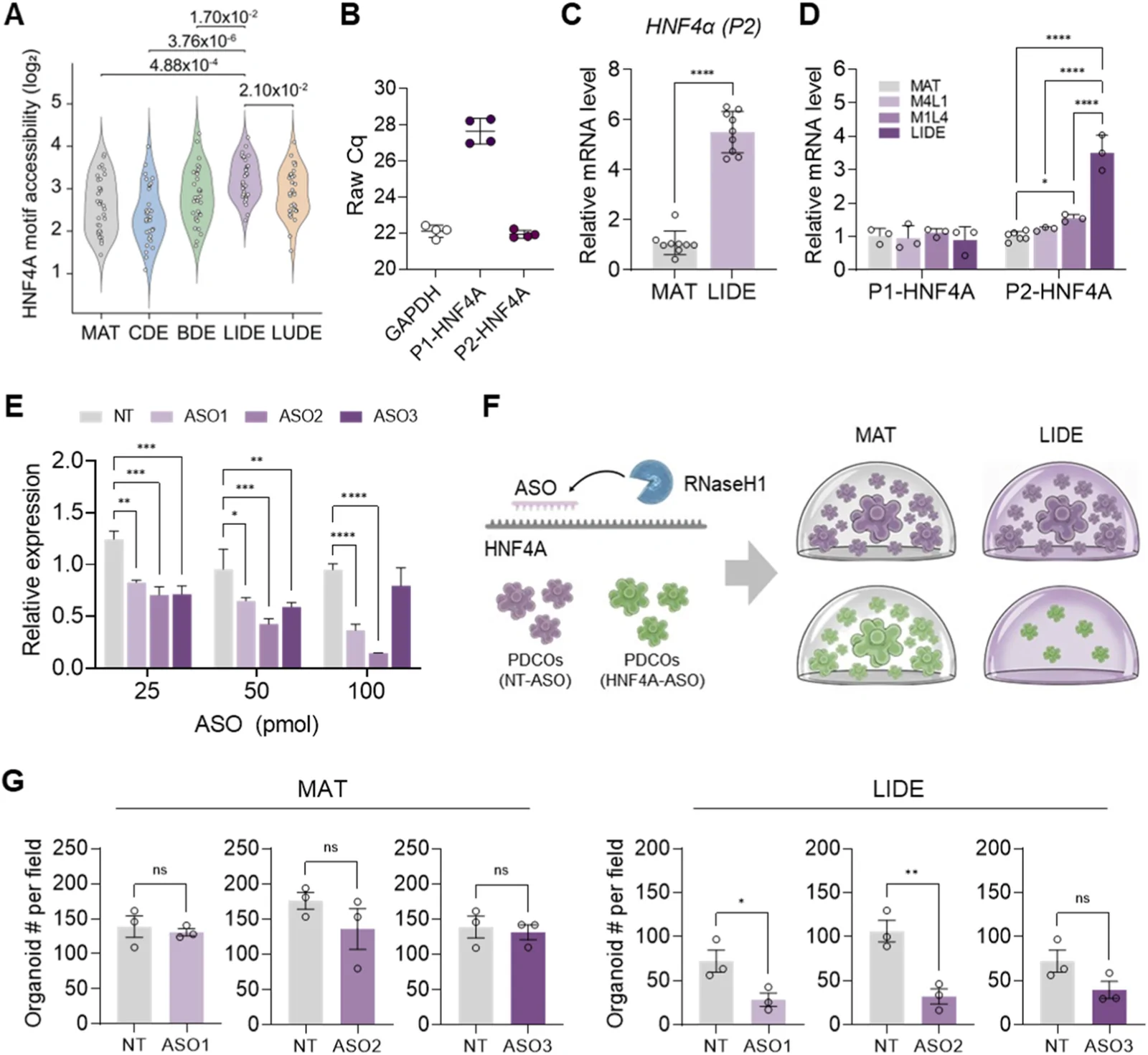
**HNF4A depletion impairs adaptation of PDCOs to liver-derived dECM (LIDE).** (A) Distribution of chromatin accessibility of LIDE day 2-enriched HNF4A motif score across different hydrogel conditions. The p-value is tested using Wilcoxon and Benjamini-Hochberg adjusted. (B) Isoform-specific quantitative PCR analysis of *HNF4A* transcripts in PDCOs. (C) Expression of P2-*HNF4A* mRNA in PDCOs cultured on LIDE for 8 days. (D) Dose-dependent changes in P2-*HNF4A* mRNA expression in PDCOs cultured with increasing concentrations of LIDE, including M4L1 (4:1 MAT:LIDE) and M1L4 (1:4 MAT:LIDE) conditions. Data are presented as mean ± SD (n ≥ 3). Statistical significance was determined by one-way ANOVA followed by Newman-Keuls multiple comparisons test. (E) Dose-dependent reduction of *HNF4A* expression following electroporation with three independent LNA GapmeR ASOs targeting distinct *HNF4A* sequences (ASO1–3), relative to a non-targeting control ASO. Data are presented as mean ± SD (n = 2). Statistical significance was determined by one-way ANOVA followed by Dunnett's multiple comparisons test. (F) Schematic overview of antisense oligonucleotide (ASO)-mediated *HNF4A* knockdown in PDCOs. PDCOs were electroporated with *HNF4A*-targeting ASOs (HNF4A-ASO) or a non-targeting control ASO (NT-ASO) and subsequently embedded in LIDE and MAT for 3D culture. Organoid formation was quantified on day 8. (G) Organoid-forming efficiency of ASO-treated PDCOs cultured in LIDE and MAT. For (C) and (G), data are presented as mean ± SD (n ≥ 3), and statistical significance was determined using Student's t-test for two-group comparisons. *p < 0.05; **p < 0.01; ***p < 0.001; ****p < 0.0001.

*HNF4A* is a key regulator of hepatic gene expression essential for liver function, with disruption impairing hepatic epithelium development and liver morphogenesis [66,67]. HNF4A binding motifs were uniquely enriched in LIDE at the initial stage of adaptation (Fig. 6A), identifying *HNF4A* as a candidate regulator of PDCO adaptation within a liver ECM-like niche. Supporting the functional relevance of these accessible regions, HNF4A-associated loci subsequently gained H3K27ac by day 8 (Supplementary Fig. 7A). Interestingly, H3K27ac enrichment at these regions was also observed in CDE (Supplementary Fig. 7B). More broadly, CDE and LIDE exhibited a largely shared H3K27ac landscape relative to MAT, whereas the corresponding chromatin accessibility changes remained substantially more tissue specific (Supplementary Fig. 7C and D). These findings suggest that enhancer activation and chromatin accessibility represent complementary but distinct layers of ECM-driven epigenetic regulation [[68], [69], [70], [71], [72]].

Based on these observations, we next investigated the expression pattern and functional role of *HNF4A* during PDCO adaptation. *HNF4A* is well known to have two major isoforms expressed by P1 and P2 promoters [73]. Although reduced total *HNF4A* activity has been associated with CRC metastasis [74,75], previous studies did not distinguish promoter-specific isoforms. In our PDCOs, the oncogenic P2-*HNF4A* isoform predominated (Fig. 6B) [76], whereas the tumor-suppressive P1 isoform remained low [77]. P2-*HNF4A* was significantly upregulated in PDCOs cultured in LIDE (Fig. 6C) and increased dose-dependently with increasing proportions of LIDE in MAT (Fig. 6D), indicating that P2-*HNF4A* activation is induced by liver ECM components.

To determine whether *HNF4A* is required for the survival and formation of PDCOs under liver-mimicking conditions, antisense oligonucleotide (ASO) targeting *HNF4A* was delivered into organoids via electroporation (Fig. 6E), followed by embedding in MAT or LIDE hydrogels (Fig. 6F). Organoid-forming efficiency was markedly reduced in LIDE upon *HNF4A* knockdown, whereas no significant change was observed in MAT (Fig. 6G), indicating that HNF4A is essential for organoid development in a liver-like ECM environment.

We next asked whether *HNF4A* activation alone could substitute for liver-derived ECM cues. PDCOs overexpressing either P1- or P2-*HNF4A* were cultured in collagen type I hydrogels lacking liver-derived ECM cues (Supplementary Fig. 8A). Neither isoform enhanced organoid-forming efficiency (Supplementary Fig. 8B), indicating that HNF4A activation alone is insufficient to support PDCO adaptation in the absence of liver-derived ECM signals.

Together, these gain- and loss-of-function studies establish *HNF4A* as a necessary regulator of PDCO adaptation to liver-derived ECM. This observation is consistent with previous studies, including single-cell epigenomic analyses demonstrating HNF4A motif enrichment in CRC liver metastases compared with primary tumors [78], as well as transcriptomic and proteomic studies reporting elevated *HNF4A* expression in metastatic CRC patients [79]. Collectively, these findings support a model in which HNF4A-mediated transcriptional regulation cooperates with liver-derived ECM cues to facilitate tumor cell adaptation within the metastatic niche.

### 2.7 Integrin and MET signaling contribute to early adaptive responses of PDCOs in LIDE

**Fig. 7:**
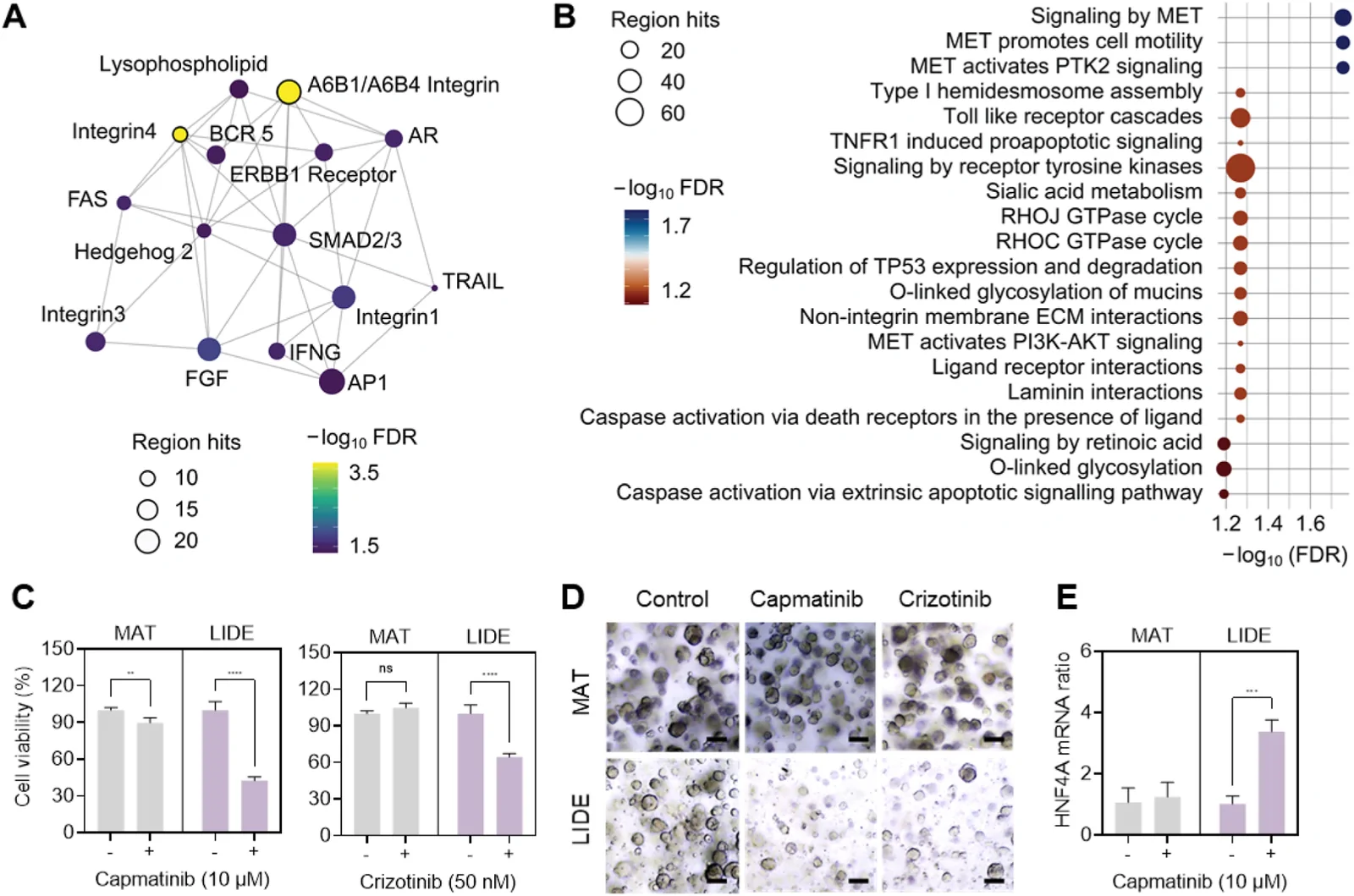
**Pathway enrichment analysis and functional validation of liver-derived dECM (LIDE)-specific chromatin programs.** (A) Network visualization of LIDE-accessible peaks at day 2 (peak groups 1 and 3 in Fig. 5H) associated enriched pathways from the MSigDB C2:PID collection. Node size represents the number of contributing regions, and node color indicates −log10 transformed FDR. Edges denote pathway overlap based on shared genes. (B) Dot plot of enriched pathways from the MSigDB C2:Reactome collection for the same LIDE-specific peak subset used in Fig. 7A. Dot size corresponds to the number of region hits, and color indicates −log10(FDR). (C) Cell viability assessment of PDCOs cultured in LIDE following treatment with MET inhibitors. (D) Representative organoid images after treatment with 10 μM capmatinib or 50 nM crizotinib are shown on the right. Scale bar, 100 μm. (E) P2-*HNF4A* expression in PDCOs cultured in LIDE following treatment with the MET-specific inhibitor capmatinib. In (A) and (B), FDR values are hypergeometric test p-values adjusted for multiple testing by the Benjamini–Hochberg procedure. Statistical significance in (C) and (E) was determined by one-way ANOVA followed by Tukey's post hoc test for multiple comparisons. *p < 0.05; **p < 0.01; ***p < 0.001; ****p < 0.0001.

To investigate extracellular signaling pathways induced by LIDE, we performed pathway enrichment analysis of early LIDE-accessible chromatin regions (peak groups 1 and 3 in Fig. 5H), which highlighted integrin-associated signaling (such as integrin α4 and α6β1/α6β4) (Fig. 7A) and MET signaling (Fig. 7B), consistent with enhanced cell–ECM interactions in collagen-rich dECM compared with glycoprotein-dominant MAT (Fig. 2E).

We next assessed the functional relevance of integrin signaling during LIDE adaptation by siRNA-mediated perturbation of candidate integrin receptors (Supplementary Fig. 10A). Among the candidates, *ITGB1* depletion produced the most consistent reduction in organoid formation in both MAT and LIDE hydrogels, with a greater effect in LIDE (Supplementary Fig. 10B) and decreased P2-*HNF4A* expression in LIDE-cultured PDCOs (Supplementary Fig. 10C). These findings are consistent with previous reports showing that *ITGB1* promotes CRC progression and liver metastasis by enhancing tumor cell migration and invasion [80,81]. To gain additional mechanistic insight into *HNF4A* regulation, we next examined STAT3, which can be active downstream of ECM-associated receptor pathways, including integrin signaling [82]. Pharmacological inhibition of STAT3 reduced P2-*HNF4A* expression in LIDE-cultured PDCOs (Supplementary Fig. 9), suggesting that STAT3-associated signaling may contribute to *HNF4A* regulation during PDCO adaptation to liver-derived ECM environment.

Given that MET signaling was also identified as an enriched pathway during early LIDE adaptation and has been implicated in tumor cell survival and anchorage-independent growth [83], we next examined the functional relevance of MET signaling in LIDE adaptation. PDCOs cultured in LIDE were treated with the c-MET inhibitors capmatinib and crizotinib (Fig. 7C and D). PDCOs cultured in LIDE exhibited a significant reduction in viability upon c-MET inhibition, whereas those cultured in MAT showed minimal sensitivity across the tested doses. These results indicate that PDCO survival in LIDE is dependent on MET-associated signaling. Interestingly, c-MET inhibition under LIDE conditions led to increased expression of P2-*HNF4A* (Fig. 7E), suggesting that blockade of MET signaling may elicit an alternative adaptive transcriptional response in PDCOs.

Together, these findings suggest that early adaptation of PDCOs to liver-derived ECM involves multiple signaling pathways rather than a single dominant regulator. β1-integrin and c-MET contribute to PDCO survival within the liver ECM environment, whereas STAT3-associated signaling may contribute to *HNF4A* regulation. The increase in P2-*HNF4A* following c-MET inhibition further highlights the transcriptional plasticity associated with early metastatic adaptation. These findings provide a basis for future studies to define how MET and other ECM-associated pathways converge on *HNF4A* regulation during early metastatic adaptation.

Consistent with the ECM-induced c-MET dependency observed in our study, previous studies have demonstrated that suppression of c-MET reduces hepatic metastatic nodule formation of CRC cells *in vivo* [84], and that c-MET inhibition limits metastatic outgrowth in other solid tumors [85,86]. Collectively, these findings support the therapeutic relevance of targeting c-MET during metastatic adaptation. Future studies using orthotopic or intrasplenic CRC models will be important to determine whether the ECM-dependent c-MET vulnerability identified here similarly limits metastatic colonization and outgrowth *in vivo*.

### 2.8 Conclusion

This study establishes tissue-specific dECM hydrogels as active biomaterials that instruct cancer organoid behavior by preserving organ-specific biochemical cues. In PDCOs, CDE outperformed Matrigel in maintaining stemness-associated features and colon-specific epigenetic states, underscoring its value for faithful modeling of primary tumors. Extending this platform to secondary tissue environments, we show that brain-, liver-, and lung-derived ECMs differentially regulate organoid growth, cellular states, and chromatin accessibility, demonstrating that ECM composition alone is sufficient to reshape transcriptional regulatory programs in metastatic cancer models.

Conceptually, this platform captures an early metastatic niche–sensing phase in which disseminated tumor cells encounter unfamiliar ECM environments and undergo rapid epigenetic adaptation prior to stable colonization. Because clinical samples and *in vivo* models predominantly represent established metastatic lesions, this early adaptive phase remains difficult to study. By recreating tissue-specific ECM environments, our system allows for the examination of the role of extracellular biochemical composition in epigenetic state transitions. The early enrichment of stimulus-responsive and tissue-specific regulators (Fig. 5L and 6A) demonstrates that this platform captures an active phase of ECM-driven adaptation, although earlier sampling will be required to resolve the primary cis-regulatory events that initiate these responses.

Within this framework, LIDE uniquely engaged *HNF4A*-associated regulatory programs and rendered PDCOs selectively dependent on c-MET signaling. Loss-of-function studies demonstrated that *HNF4A* is required for early PDCO adaptation in LIDE, while pharmacological inhibition of c-MET selectively impaired PDCO viability under liver-mimetic conditions. Consistent with prior studies in solid tumors, c-MET activation promotes tumor cell survival, proliferation, and stemness [87]. In line with previous findings, PDCOs cultured in LIDE exhibited partially increased expression of stem cell markers (Fig. 5F), with altered chromatin accessibility before transcription. While the precise molecular hierarchy linking ECM composition to MET dependency remains to be fully delineated, our data support a model in which liver-derived ECM cues prime selective signaling vulnerabilities during early adaptation. Furthermore, c-MET inhibition under LIDE conditions increased *HNF4A* expression, suggesting that disruption of MET-associated survival signaling can unmask alternative lineage-linked transcriptional programs. Together, these observations highlight the dynamic interplay between ECM-derived signals and transcriptional plasticity during early metastatic adaptation, imposing organ-selective adaptive constraints on disseminated tumor cells. Extending these findings to long-term *in vivo* metastasis models will clarify whether ECM-driven transcriptional plasticity governs metastatic colonization, organ tropism, and the persistence of disseminated tumor cells.

More broadly, the conceptual framework described here may extend beyond colorectal cancer. Because metastatic adaptation is a common challenge across many solid tumors, this strategy could be extended to patient-derived organoids from other malignancies, including pancreatic, breast, gastric, lung, prostate, and urological cancers, by reconstructing their corresponding tissue-specific metastatic microenvironments. Such adaptability highlights the broad potential of tissue-derived dECM platforms for studying organ-selective metastatic colonization across diverse cancer types.

Beyond its broad applicability, this platform also provides new opportunities to address fundamental questions in metastatic adaptation. Sequential ECM-transfer studies could reveal whether ECM-induced adaptive states are reversible or maintained through epigenetic memory [88], whereas incorporation of stromal, immune, and vascular components into multicellular co-culture systems will enable investigation of metastatic adaptation within more physiologically relevant tissue niches [[89], [90], [91], [92], [93]].

Furthermore, integrating component-resolved ECM analysis with single-cell epigenomics and patient-derived organoids cultured in matched patient-derived dECM hydrogels could enable systematic identification of organ-specific metastatic vulnerabilities while establishing personalized platforms for therapeutic evaluation and drug sensitivity testing. Ultimately, this biomaterial-guided platform may provide a foundation for tailored treatment evaluation, identification of ECM-dependent metastatic vulnerabilities, and development of anti-metastatic therapeutic strategies.

## 3 Materials and methods

### 3.1 Decellularization of porcine tissues

To obtain each organ tissue-derived decellularized ECM, liver and lung tissue from the porcine were purchased in a local market, and brain from the porcine collected from local slaughterhouse. The tissues were immediately frozen and stored at −80 °C until use. The decellularization protocols for each tissue were performed according to previously reported methods with minor modifications [[39], [40], [41]].

For the brain tissue decellularization, the tissues were thawed at room temperature and cut into approximately <1 cm^3^ pieces. The tissue pieces were immersed in pre-cooled distilled water (DW) and washed for 24 h at 4 °C with continuous agitation, during which the DW was replaced several times to facilitate the removal of blood and soluble components. The tissues were then sequentially incubated at room temperature in 0.02% trypsin/ethylenediaminetetraacetic acid (EDTA) solution (Welgene, Republic of Korea) for 2 h, 3% Triton X-100 solution (LPS solution, Republic of Korea) for 1 h, 1 M sucrose solution (Sigma-Aldrich, USA) for 45 min, and 4% sodium deoxycholate solution (Sigma-Aldrich, USA) for 1 h, with each incubation step followed by washing with pre-cooled Dulbecco's phosphate-buffered saline (DPBS) at 4 °C. After decellularization, the tissues were thoroughly washed with pre-cooled DPBS at 4 °C, replacing the solution several times to remove residual detergents, and subsequently incubated in 1% penicillin-streptomycin (P/S) solution (Welgene, Republic of Korea) for 24 h under refrigerated conditions for sterilization.

For the liver tissue decellularization, the frozen liver tissues were cut into approximately < 0.5 cm^3^ pieces and washed with pre-cooled DW for 12 h at 4 °C, during which the DW was replaced several times, to remove residual blood and tissue debris. Following the initial washing step, the tissues were immersed in 0.5% Triton X-100 solution overnight at 4 °C for detergent-mediated cell lysis. After Triton X-100 treatment, the tissues were thoroughly rinsed with pre-cooled DW to remove residual detergent. The tissues were subsequently incubated in 0.1% sodium dodecyl sulfate (SDS) solution (Sigma-Aldrich, USA) for 24 h at 4 °C to further eliminate cellular components and were then rinsed again with pre-cooled DW. The decellularized tissues were sterilized by incubation in 1% P/S solution for 24 h. Finally, the tissues were washed with DW to remove residual antibiotics prior to subsequent experiments.

For the lung tissue decellularization, the frozen lung tissues were cut into approximately < 1 cm^3^-sized pieces. And the tissues were washed with pre-cold DW for 12 h at 4 °C, with the DW replaced several times to remove residual blood and tissue debris. After washing the tissues, the tissues were immersed in 0.1% Triton X-100 solution for 72 h at 4 °C, followed by thorough washing with pre-cold DW to remove the residual detergent. The washed tissues were subsequently immersed in 1% SDS solution and incubated for 24 h at room temperature, followed by another thorough wash with pre-cold DW. After completion of the decellularization process, the tissues were incubated in 1% P/S solution for 24 h for sterilization. Finally, the tissues were washed with DW to remove residual antibiotics prior to subsequent experiments.

Decellularization of fresh porcine colon tissue was performed to obtain lyophilized colon dECM, as described in a previous protocol [38]. Briefly, freshly frozen, food-grade porcine colon tissue was purchased from an online commercial butchery. After thawing, the colon tissue was washed, and the submucosal layer was physically separated and cut into appropriate sizes to maximize the effectiveness of subsequent chemical and enzymatic reactions for the removal of cellular components and impurities. During trimming, the tissues were immersed in DW to prevent dehydration. The trimmed submucosal tissues were sequentially treated with ethyl alcohol, penicillin/streptomycin, SDS, EDTA, Triton X-100, isopropyl alcohol, and deoxyribonuclease. Following the removal of cellular and lipid components, the acellular tissues were sterilized using a peracetic acid/ethanol solution. All solutions were prepared fresh before use.

Finally, all the decellularized tissues were snap-frozen, lyophilized (ilShinBioBase, Republic of Korea), and stored at −80 °C.

### 3.2 Preparation of the dECM solution

The lyophilized ECM was resuspended in a 0.01 N HCl solution (Welgene, Republic of Korea) at a concentration of 10 mg/mL and digested with 1 mg/mL pepsin (Sigma-Aldrich, USA) for 48 h at room temperature, whereas for the CDE preparation, lyophilized colon dECM was hydrolyzed with pepsin in acetic acid under stirring. Following digestion, undigested residues were filtered out through a 100-μm strainer (SPL Life Sciences, Republic of Korea). The digest was then neutralized to a pH of 7.2–7.5 using 10X phosphate-buffered saline (PBS, Gibco, USA) and 0.1 M sodium hydroxide solution (Sigma-Aldrich, USA) while maintaining 4 °C. The resulting 10 mg/mL dECM solution was cryopreserved at −20 °C until used for future experiments. To form the final dECM-based hydrogel, each dECM solution was first diluted to the desired concentration with Advanced DMEM/F12 (Gibco, USA), followed by incubation at 37 °C for 30 min to induce gelation.

### 3.3 Proteomic analysis of dECM

E-biogen (E-biogen, Inc., South Korea) was commissioned to perform the proteomics. Protein concentration was determined using the Pierce BCA Protein Assay Kit (Thermo Scientific, USA). The digestion process was executed using a Filter-Aided Sample Preparation (FASP) on a Microcon 30K centrifugal filter device (Millipore, USA). Samples were first reduced by incubating with Tris(2-carboxyethyl)phosphine at 37 °C for 30 min, followed by alkylation with iodoacetic acid (IAA) at 25 °C for 1 h in the dark. After sequential washing with lysis buffer and 50 mM Ammonium Bicarbonate, proteins were digested with trypsin (with a 1:50 enzyme to protein ratio, w/w) at 37 °C for 18 h. Resulting peptides were transferred to new tubes, with trypsin inactivation achieved by adding 14 μL of formic acid (Honeywell, USA). The peptides were then desalted using C18 spin columns (Harvard Apparatus, USA), with elution achieved using 80% acetonitrile in 0.1% formic acid in water.

The prepared samples were resuspended in 0.1% formic acid in water and analyzed using a Q-Exactive Orbitrap hybrid mass spectrometer (Thermo Scientific, USA) along with an Ultimate 3000 system (Thermo Scientific, USA). We used a 2 cm × 75 μm ID trap column packed with 3 μm C18 resin and a 50 cm × 75 μm ID analytical column packed with 2 μm C18 resin to peptides depending on the peptides’ hydrophobicity. The mobile phase solvents consisted of (A) 0.1% formic acid in water and (B) 0.1% formic acid in 90% acetonitrile, and the flow rate was fixed at 300 nL min-1. The gradient of mobile phase was as follows: 4% solvent B in 14 min, 4–15% solvent B in 61 min, 15–28% solvent B in 50 min, 28–40% solvent B in 20 min, 40– 96% solvent B in 2 min, holding at 96% of solvent B in 13 min, 96–4% solvent B in 1 min, and 4% solvent B for 24 min. A data-dependent acquisition method was adopted, and the top 10 precursor peaks were selected and isolated for fragmentation. Ions were scanned in high resolution (70,000 in MS1, 17,500 in MS2 at *m*/*z* 400), and the MS scan range was 400–2000 *m*/*z* at both the MS1 and MS2 levels. Precursor ions were fragmented with Normalized Collisional Energy 27%. Dynamic exclusion was set to 30 s.

Thermo MS/MS raw files of each analysis were searched by using Proteome Discoverer™ software (ver. 2.5), and the *Sus scrofa* database was downloaded from UniProt. The appropriate consensus workflow included a peptide-spectrum match validation step and SEQUEST HT process for detection as a database search algorithm. The search parameters were set up as follows: 10 ppm of tolerances of precursor ion masses, 0.02 Da fragment ion mass, maximum of two missed cleavages with trypsin enzyme. After searching, the data results below 1% of FDR were selected and filtered at least 6 more peptides length. The mass spectrometry proteomics data have been deposited to the ProteomeXchange Consortium via the PRIDE 27 partner repository with the dataset identifier PXD065242.

### 3.4 Characterization of dECM

To quantify the amount of DNA remaining in each native and dECM, DNA content was measured using an AccuPrep Genomic DNA extraction kit (Bioneer, Republic of Korea) following the manufacturer's instructions. The concentration of the DNA was measured using a NanoDrop™ One spectrophotometer (Thermo Scientific, USA) at 260 nm.

Total hydroxyproline content of both native tissue and dECM was quantified with hydroxyproline assay kit (Biomax, Republic of Korea). The glycosaminoglycan content was measured with a dimethylmethylene blue assay kit (Blyscan, USA). All assay kits were used in accordance with each manufacturer's guidelines.

For the histological evaluation, the samples were fixed with 4% paraformaldehyde solution and embedded in paraffin. Tissue sections (10 μm) were stained with hematoxylin and eosin (H&E), following standard procedures.

Rheological measurements were performed using a HAAKE MARS III rheometer (Thermo Scientific, USA). To determine the linear viscoelastic region, an oscillatory amplitude sweep was conducted with a strain range of 0.01–10% at a fixed frequency of 0.1 Hz. To analyze the viscoelastic properties of each dECM-based hydrogel as a function of frequency, an oscillatory frequency sweep was performed at a constant strain of 0.5% over a frequency range of 0.05–100 rad/s.

### 3.5 PDCO establishment and maintenance

The use of patient tissue samples and clinical data was approved by the Institutional Review Board of Asan Medical Center (IRB No. 2019-0340), while approval for the generation and use of PDOs was granted by the Institutional Review Board of UNIST (IRB No. UNISTIRB-18-49-A).The PDCOs were established from the resected CRC tissue segments obtained from Asan Medical Center as previously described [94].

The established PDCOs were embedded in growth factor reduced Matrigel (Corning, USA) and cultured in Advanced DMEM/F12 (Gibco, USA) supplemented with 10% R‐spondin conditioned media (CM), 5% Fetal bovine serum (Gibco, USA), 1X B27 (Gibco, USA), 10 mM HEPES (Welgene, Republic of Korea), 2 mM GlutaMax (Gibco, USA), 100 μg/mL Primocin (InvivoGen, USA), 5 μg/mL Plasmocin (InvivoGen, USA), 10 mM Nicotinamide (Sigma‐Aldrich, USA), 1 mM N‐acetyl-L-cysteine (Sigma‐Aldrich, USA), 100 ng/mL Noggin (PeproTech, USA), 10 μM Y‐27632 (Selleckchem, USA), 10 μM SB202190 (BioGems, USA), 50 ng/mL Human epidermal growth factor (PeproTech, USA), 500 nM A83‐01 (Tocris, UK), 10 nM Prostaglandin E2 (PeproTech, USA) and 10 nM Gastrin (Sigma-Aldrich, USA). The culture medium was refreshed every 3–4 days. For each passage (performed every 8–10 days), organoids were mechanically dissociated by vigorous pipetting, separated from the residual matrix, enzymatically singularized using TrypLE (Gibco, USA), centrifuged to form a pellet, and subsequently reseeded in fresh MAT. HA-R-spondin 1-Fc 293T cells (Trevigen, cat no. 3710-001-K) were used to produce R-spondin-1 CM and cultured according to the manufacturer's protocol.

### 3.6 PDCO culture in dECM hydrogel

Organoid singularization was performed with slight modifications from a previously described method [95]. Briefly, organoids were first collected using cold DPBS, then incubated with TrypLE Express (Gibco, USA) at 37 °C for 5 min. The reaction was quenched by adding media containing FBS, followed by centrifugation. The pellet was resuspended in fresh media for subsequent experiments. Singularized PDCOs were adjusted to a concentration of 0.8-1.0 × 10^6^ cells/mL and mixed in a 1:1 ratio with either colon brain, liver, or lung dECM solutions at a concentration of 10 mg/mL. This cell/dECM mixture was then plated onto a cell culture plate in dome-shaped droplets of 20 μL, depending on the experimental purpose. The plate was incubated in a humidified CO_2_ incubator at 37 °C for 30–45 min to allow the formation of a cell-laden hydrogel.

### 3.7 Organoid-forming assessment

The singularized organoid cells were embedded in each dECM-based hydrogel and MAT. Subsequently, the cells were incubated in a humidified CO_2_ incubator at 37 °C for 8 days, and the medium was changed every 3–4 days. After cultivation, images of the formed organoids were captured using a brightfield microscope. More than five images were obtained per dome. These images were analyzed using ImageJ software (National Institutes of Health, USA) to count and quantify the number of organoids formed per field, as well as to measure their diameter and size distribution.

### 3.8 Cell viability assay

The effect of 3D culture on the cell proliferation was assessed using water soluble tetrazolium salt WST-8 (Quanti-Max™ WST-8 Cell Viability Assay Kit, Biomax, Republic of Korea). PDCOs encapsulated in each dECM-based hydrogel and MAT were additionally incubated for 2 h with 10% WST-8 reagent at each predetermined time point. Then, the absorbance was measured at 450 nm using a microplate reader (BioTek, USA). The cell proliferation rate was calculated relative to day 2.

Cell viability of PDCOs cultured in each hydrogel following inhibitor treatment was assessed using the CellTiter-Glo® 3D Cell Viability Assay (Promega, USA) according to the manufacturer's instructions. Briefly, an equal volume of CellTiter-Glo® 3D reagent was added to each well containing PDCOs and culture medium. The plate was gently mixed to induce cell lysis and incubated at room temperature for 10 min to stabilize the luminescent signal. Luminescence intensity, which is proportional to intracellular ATP levels, was measured using a microplate reader (BioTek, USA).

To evaluate the LDH release as an indicator of cytotoxicity, the culture supernatants of PDCOs in each decellularized ECM-based hydrogel were collected and analyzed with CytoTox 96 Non-Radioactive Cytotoxicity Assay (Promega, USA) following the manufacturer's protocol.

### 3.9 qPCR analysis

Organoids were harvested from each decellularized ECM-based hydrogel and MAT as mentioned before. Total RNA of organoids was extracted with TRIzol (Invitrogen, USA) following the manufacturer's instructions. The RNA concentration was quantified using a Nanodrop™ One spectrophotometer (Thermo Scientific, USA). 500 ng of extracted RNA was converted to cDNA using AccuPower RocketScript RT PreMix (Bioneer, Republic of Korea). qPCR was conducted using SYBR® Green Realtime PCR Master Mix (TOYOBO, Japan). Relative quantification of target genes was performed using the 2−ΔΔCT method. First, the target gene in each sample was normalized by *RPS27* as a reference gene based on Ct values. The fold changes in gene expression were analyzed relative to the control group (PDCO cultured in MAT). The primers used in this study are listed in Supplementary Table 1.

### 3.10 Immunofluorescence staining

PDCOs were collected in chilled cell recovery solution (Corning, USA) and incubated for 45 min on ice to completely dissolve remaining MAT. For decomposition of dECM hydrogel, collagenase type I was applied at 37 °C for 10 min. After incubation, the PDCOs were collected and washed with DPBS. Then, organoids were fixed with 4% paraformaldehyde solution and permeabilized with 0.2% Triton X-100 solution (LPS solution, Republic of Korea) in DPBS. The cells were then treated with 3% bovine serum albumin (Georgiachem, USA) in DPBS with 0.02% Triton X-100 for 1 h at RT to block the non-specific binding of antibodies. Samples were incubated with the indicated primary antibodies—E-cadherin (Proteintech, USA), ZO-1 (Invitrogen, USA), Ki-67 (Santa Cruz Biotechnology, USA), LGR5 (Proteintech, USA) and ALDH (Santa Cruz Biotechnology, USA)—at 4 °C for 24 h. After washing, samples were incubated with Alexa Fluor 488-, 555-, 594- and 647–conjugated secondary antibodies (Invitrogen, USA). F-actin was visualized by Alexa647-conjugated phalloidin (Invitrogen, USA). Nuclei were stained with Hoechst 33342 (Invitrogen, USA). Confocal imaging was performed using a Zeiss LSM 980 confocal laser scanning microscope and analyzed using the Zeiss Zen Blue software and ImageJ v1.54.

### 3.11 ATAC-seq analysis of PDCOs

For the ATAC-seq analysis, the organoids were cultured for the desired periods (day 2, 4 and 8) and singularized by the method mentioned in 4.2. Subsequently, singularized organoid cells were washed with cold PBS twice, and cells displaying apoptotic signals were discarded using Annexin V MicroBead Kit (Miltenyi Biotec, 130-090-201), following the manufacturer's instructions. Thereafter, viable cells were collected and utilized for ATAC-seq according to the protocol described by Corces et al. [96] with some modifications.

Briefly, 50,000 viable cells were pelleted and resuspended in 50 μL of ATAC-seq resuspension buffer (RSB; 10 mM Tris-HCl pH 7.4, 10 mM NaCl, 3 mM MgCl_2_) supplemented with 0.1% NP-40, 0.1% Tween-20, and 0.01% digitonin, and lysed on ice for 3 min. Lysis was quenched with RSB containing 0.1% Tween-20, and nuclei were pelleted (500 × g, 10 min, 4 °C). Nuclei were resuspended in 50 μL of transposition mix (25 μL 2× TD buffer, 2.5 μL Tn5 transposase, 16.5 μL PBS, 0.5 μL 1% digitonin, 0.5 μL 10% Tween-20, and 5 μL water) and tagmented at 37 °C for 60 min with shaking (1000 r.p.m.). Tagmented DNA was purified using the DNA Clean & Concentrator-5 (Zymo, D4004). Libraries were amplified using Nextera indexing primers, with the cycle number for each library determined individually by qPCR-based monitoring to avoid over-amplification, purified with the DNA Clean & Concentrator-5, and paired-end sequenced on an Illumina NovaSeq system.

Raw reads were quality-checked with FastQC v0.11.9 and adapter-trimmed using in-house adapter trimming script, which removes Nextera adapter sequences. Trimmed reads were aligned to hg38 with Bowtie2 v2.4.4, and PCR duplicates (picard MarkDuplicates v3.0.0), mitochondrial reads, low-mapping-quality reads (MAPQ < 30), and reads overlapping ENCODE hg38 blacklist regions were removed. Library quality was verified by the expected nucleosomal fragment-size periodicity, transcription start site (TSS) enrichment (TSS score > 3.0); all retained libraries met ENCODE ATAC-seq quality standards. Peaks were called using MACS2 v2.2.7.1 ("-f BAMPE -g hs --nomodel --shift −100 --extsize 200″), and reproducibility between the two biological replicates was confirmed by Pearson correlation of genome-wide accessibility profiles. Genomic annotation of called peaks was performed using ChIPseeker (1.42.1).

For the direct MAT-versus-CDE comparison, differential accessibility was quantified on the consensus peak count matrix using DESeq2 (1.46.0). To identify progressive and condition-specific accessibility changes across multiple time points, we applied the distal binarization approach described previously by Corces et al. [58]. Using the binarized matrix (|FC| > 2, FDR < 0.1), we identified peaks showing progressively increased accessibility from day 2 to day 4 to day 8, as well as peaks uniquely accessible at individual time points (days 2, 4, or 8), for downstream analyses.

To assess the cancer-type specificity of ECM-induced accessibility changes, the accessibility of these differentially accessible regions and the motif-containing feature peaks (e.g., CDX1-motif peaks) was evaluated across the pan-cancer ATAC-seq dataset [58], which comprises 410 tumor samples spanning 23 cancer types in The Cancer Genome Atlas (TCGA). Publicly available processed accessibility matrices provided in the supplemental data of Corces et al. were used, and all cancer types were included in the analysis. To identify tissue-specific accessibility changes across BDE, LIDE, and LUDE, the previously described distal binarization approach was applied using more permissive thresholds (|FC| > 1.5, FDR < 0.1), with MAT as the common reference condition representing the shared day 0 baseline for all culture conditions prior to ECM-induced chromatin remodeling.

Next, to identify enriched transcription factor binding motifs within peak clusters, we utilized non-redundant transcription factor motifs [97]. The archetype MEME models (v2.0) were converted to position weight matrix format for compatibility with motifmatchr (v1.28.0). Enrichment of each transcription factor was determined using the hypergeometric test p-value, with all motifs serving as background.

### 3.12 H3K27ac CUT&Tag analysis of PDCOs

For H3K27ac CUT&Tag, viable organoids were prepared as described for the ATAC-seq analysis (Section 3.11). For each biological replicate, 100,000 viable cells were collected for each antibody (H3K27ac, Abcam #ab4729; IgG negative control, Cell Signaling Technology #2729). Libraries were prepared using the CUT&Tag Assay Kit (Cell Signaling Technology #77552S).

Reads were quality-checked with FastQC, adapter-trimmed as for ATAC-seq, and aligned to hg38 using Bowtie2 v2.4.4 (--local --very-sensitive-local --no-mixed --no-discordant -I 10 -X 700). Alignments with MAPQ < 30 and reads overlapping ENCODE hg38 blacklist regions were discarded. Peaks were called with MACS2 v2.2.7.1 (narrowPeak mode, matched IgG as control, q < 0.01) and annotated with ChIPseeker v1.42.1. FRiP values exceeded 0.4 for all H3K27ac libraries and were below 0.1 for matched IgG controls; replicate reproducibility was confirmed by Pearson correlation of genome-wide H3K27ac signal.

H3K27ac enrichment was quantified over the ATAC-seq consensus peak set and correlated with chromatin accessibility on a per-peak basis. Enrichment at motif-containing regions (500-bp windows, motif ± 250 bp) was compared with peak-strength–matched background windows selected by nearest-neighbor matching (K = 50) on DESeq2-normalized mean H3K27ac signal (baseMean) across all replicates and conditions. Over 5000 permutations, one matched background window per motif was sampled to build a null distribution of median log_2_ fold changes, from which one-sided empirical P values were derived.

### 3.13 ASOs and siRNAs transfection in PDCOs by electroporation

Antisense oligonucleotides (ASOs) targeting HNF4A were designed with the QIAGEN GeneGlobe design tool and synthesized as antisense LNA GapmeRs (QIAGEN): HNF4A-ASO1 (LG00846535-DDA), HNF4A-ASO2 (LG00846534-DDA), and HNF4A-ASO3 (LG00846528-DDA). Antisense LNA GapmeR Negative Control A (LG00000002-DDA) was electroporated at matched amounts as a non-targeting control. ASOs were used at 100 pmol per 1 × 10^6^ cells (10 μM final) unless otherwise indicated. All ASO sequences and product identifiers are listed in Supplementary Table 2.

siRNAs targeting integrins and syndecans were HPLC-purified AccuTarget™ Genome-wide Predesigned siRNAs (BIONEER, cat. no. SDH-1001), with three independent duplexes targeting distinct regions of each transcript. A GAPDH-targeting siRNA and a non-targeting negative control siRNA (AccuTarget™ GAPDH Control siRNA Set; BIONEER, cat. no. SS-1011) served as positive and negative controls, respectively. 100 pmol of siRNA was delivered per 1 × 10^6^ cells. All siRNA sequences and product identifiers are listed in Supplementary Table 3.

PDCOs were dissociated into single cells as described, and 1 × 10^6^ cells were resuspended in 10 μL of Resuspension Buffer R (Invitrogen, MPK1096). Oligonucleotide was added to the suspension, which was then loaded into a 10 μL Neon™ tip (Invitrogen, MPK1096) and electroporated on a Neon™ Transfection System (Invitrogen, MPK5000S) using two 20 ms pulses at 1350 V.

Gene expression levels were quantified by qPCR following RNA extraction using TRIzol, using primers in Supplementary Table 1.

### 3.14 Statistical analysis

All quantitative data are expressed as the mean ± standard deviation (SD). Statistical analyses were performed using Student's t-test for comparisons between two groups. One-way or two-way ANOVA with appropriate multiple comparison tests was used for comparisons among multiple groups. All analyses were conducted using GraphPad Prism software (version 8, GraphPad Software, Inc., San Diego, CA, USA; *P < 0.05; **P ≤ 0.01; ***P ≤ 0.001; ****P ≤ 0.0001).

### 3.15 Data availability

The ATAC-seq and H3K27ac CUT&Tag data generated in this study have been deposited in the NCBI Gene Expression Omnibus (GEO) under accession number GSE324972. All other data supporting the findings of this study are available within the article and its Supplementary Information, or from the corresponding author upon reasonable request.

## CRediT authorship contribution statement

**Heejeong Yoon:** Writing – original draft, Visualization, Methodology, Investigation, Data curation, Conceptualization. **Dayoung Kim:** Writing – original draft, Visualization, Methodology, Investigation, Data curation, Conceptualization. **Hye-Jin Jeong:** Investigation. **Hohyeon Han:** Resources. **Seon-Jin Kim:** Resources. **Jinah Jang:** Resources. **Deok-Beom Jung:** Resources. **Seung-Jae Myung:** Resources. **Chang Sik Yu:** Resources. **In Ho Song:** Resources. **Ju Hae Choi:** Methodology. **M. Ryan Corces:** Methodology. **Taejoon Kwon:** Funding acquisition, Conceptualization. **Kyungjae Myung:** Conceptualization. **Jinmyoung Joo:** Conceptualization. **Joo H. Kang:** Funding acquisition, Conceptualization. **Tae-Eun Park:** Writing – original draft, Supervision, Funding acquisition, Conceptualization. **Seung Woo Cho:** Writing – original draft, Supervision, Funding acquisition, Conceptualization.

## Ethics approval and consent to participate

The colorectal cancer (CRC) patient-derived organoids were established from the resected CRC tissue segments obtained from Asan Medical Center. The use of patient tissue samples and clinical data was approved by the Institutional Review Board of Asan Medical Center (IRB No. 2019-0340), while approval for the generation and use of patient-derived organoids was granted by the Institutional Review Board of UNIST (IRB No. UNISTIRB-18-49-A). The case details, personal information and images of patients or any other individuals are not included in a publication.

## Declaration of competing interest

The authors declare that they have no known competing financial interests or personal relationships that could have appeared to influence the work reported in this paper.
