# Supplementary material for: Tissue-derived extracellular matrix hydrogels instruct epigenetic adaptation in metastatic colonization
Source: Bioact Mater. 2026 Aug 26;67:757–73. doi: 10.1016/j.bioactmat.2026.08.031 (PMC13544083; doi:10.1016/j.bioactmat.2026.08.031)
Supplement: Supplementary file 1 [file mmc1.pdf]

Supplementary Information

Tissue-derived extracellular matrix hydrogels instruct epigenetic adaptation in metastatic colonization

Supplementary Table 1. Primers used for qPCR

Genes	Forward (5' → 3')	Reverse (5' → 3')
ALDH1	CGCAAGACAGGCTTTTCAG	TGTATAATAGTCGCCCCCTCTC
CD166	ACTTGACGTACCTCAGAATCTCA	CATCGTCGTACTGCACACTTT
CD44	CTCTCTCCCTCCACTTCAC	TGAAAGAAACTGGACATTAGGC
CDH1	ATTTTTCCCTCGACACCCGAT	TCCCAGGCGTAGACCAAGA
GAPDH	GTCTCCTCTGACTTCAACAGCG	ACCACCCTGTTGCTGTAGCCAA
ITGA4	GGCAAGGAAGTTCCAGGTTAC	AGTTAGCTTCTCTGCTGGACA
ITGA6	TTCGAAACCAAGGTTCTGAGC	GGATCTCCACTGAGGCAGTTA
ITGB1	CCAATTGTAGCTGGTGTGGTT	GATTTTCACCCGTGTCCCATT
ITGB4	AGAGGAGACCCAGATTGACAC	TCCATGAGGATGTACAGGTCC
LGR5	GAGTTACGTCTTGCGGGAAAC	TGGGTACGTGTCTTAGCTGATTA
P1-HNF4A	*CAAAACCCTCGTCGACATGG*	*ACCGTAGTGTTTGCCCGT*
P2-HNF4A	*TCCAGTGGAGAGTTCTTACG*	*ACCGTAGTGTTTGCCCGT*
RPS	CCTTCATCCCTCTCCAGAAGA	CCGTGGTGATTTTATAGCATCCT
SDC1	TCCTGGACAGGAAAGAGGTGCT	TGTTTCGGCTCCTCCAAGGAGT
SDC2	CTGCTCAGACAAAGTCACCTG	TGCTAGGACTTCTGTCCGTTT
SDC3	CTCCTGGACAATGCCATCGACT	TGAGCAGTGTGACCAAGAAGGC
SDC4	CCCGTTGAAGAGAGTGAGGAT	TAGGATCAGGAAGACGGCAAA

Supplementary Table 2. Antisense oligonucleotides (ASO) sequences targeting HNF4A.

Name	Target gene	Sequence (5'→3')	Chemistry	Supplier	Cat. no. / product ID	Notes
Negative Control A	Non-targeting	AACACGTCTATACGC	LNA gapmer	QIAGEN	339515 / LG00000002-DDA	Non-targeting control ASO
HNF4A-ASO1	*HNF4A*	GeneGlobe custom design	LNA gapmer	QIAGEN	339515 / LG00846535-DDA	
HNF4A-ASO2	*HNF4A*	GeneGlobe custom design	LNA gapmer	QIAGEN	339515 / LG00846534-DDA	
HNF4A-ASO3	*HNF4A*	GeneGlobe custom design	LNA gapmer	QIAGEN	339515 / LG00846528-DDA	

ASO, antisense oligonucleotide; LNA, locked nucleic acid.

Supplementary Table 3. siRNAs used for gene silencing.

Name	Target gene	Sense strand (5'→3')	Antisense strand (5'→3')	Chemistry / modification	Supplier	Cat. no. / product ID
GAPDH control siRNA	*GAPDH*	Not disclosed by supplier	Not disclosed by supplier	Duplex, HPLC-purified	BIONEER	SS-1011
Negative control siRNA	Non-targeting	Not disclosed by supplier	Not disclosed by supplier	Duplex, HPLC-purified	BIONEER	SS-1011
siITGA4-1	*ITGA4*	CGAUUUACAGAUGCAGGAU=tt	AUCCUGCAUCUGUAAAUCG=tt	Duplex, HPLC-purified	BIONEER	SDH-1001 / 3676-1
siITGA4-2	*ITGA4*	CGAACCGAUGGCUCCUAGU=tt	ACUAGGAGCCAUCGGUUCG=tt	Duplex, HPLC-purified	BIONEER	SDH-1001 / 3676-3
siITGA6-1	*ITGA6*	CUCUAGGUACGAUGACAGU=tt	ACUGUCAUCGUACCUAGAG=tt	Duplex, HPLC-purified	BIONEER	SDH-1001 / 3655-1
siITGA6-2	*ITGA6*	CCAGGUGUAUUUUGGAGGU=tt	ACCUCCAAAAUACACCUGG=tt	Duplex, HPLC-purified	BIONEER	SDH-1001 / 3655-3
siITGB1-1	*ITGB1*	CAUUUGAGUUGCCCAUCUU=tt	AAGAUGGGCAACUCAAAUG=tt	Duplex, HPLC-purified	BIONEER	SDH-1001 / 3688-1
siITGB1-2	*ITGB1*	AGCCUGUUUACAAGGAGCU=tt	AGCUCCUUGUAAACAGGCU=tt	Duplex, HPLC-purified	BIONEER	SDH-1001 / 3688-3
siITGB4-1	*ITGB4*	CUGGUAAACAUCACCAUCA=tt	UGAUGGUGAUGUUUACCAG=tt	Duplex, HPLC-purified	BIONEER	SDH-1001 / 3691-1
siITGB4-2	*ITGB4*	GACGUUCUACGCUCUCCAU=tt	AUGGAGAGCGUAGAACGUC=tt	Duplex, HPLC-purified	BIONEER	SDH-1001 / 3691-3
siSDC1-1	*SDC1*	CUCCAUCCAAGGCCAGGUU=tt	AACCUGGCCUUGGAUGGAG=tt	Duplex, HPLC-purified	BIONEER	SDH-1001 / 6382-1
siSDC1-2	*SDC1*	GCUCCUGCACUUACUUGCU=tt	AGCAAGUAAGUGCAGGAGC=tt	Duplex, HPLC-purified	BIONEER	SDH-1001 / 6382-3
siSDC2-1	*SDC2*	CAGCAAACCCAAAGAUGUU=tt	AACAUCUUUGGGUUUGCUG=tt	Duplex, HPLC-purified	BIONEER	SDH-1001 / 6383-1
siSDC2-2	*SDC2*	GAAUAUCCGUGCCAGGAAA=tt	UUUCCUGGCACGGAUAUUC=tt	Duplex, HPLC-purified	BIONEER	SDH-1001 / 6383-3
siSDC3-1	*SDC3*	GACACGUACUGCAAUGCAA=tt	UUGCAUUGCAGUACGUGUC=tt	Duplex, HPLC-purified	BIONEER	SDH-1001 / 9672-1
siSDC3-2	*SDC3*	CUGCAACCUCUUGCUCGUA=tt	UACGAGCAAGAGGUUGCAG=tt	Duplex, HPLC-purified	BIONEER	SDH-1001 / 9672-3
siSDC4-1	*SDC4*	GACUUGGAAGACUCCAUGA=tt	UCAUGGAGUCUUCCAAGUC=tt	Duplex, HPLC-purified	BIONEER	SDH-1001 / 6385-1
siSDC4-2	*SDC4*	CCAGCUGGUGUUCUUUGGU=tt	ACCAAAGAACACCAGCUGG=tt	Duplex, HPLC-purified	BIONEER	SDH-1001 / 6385-3

siRNA, small interfering RNA. =tt denotes a 3' deoxythymidine dinucleotide overhang.

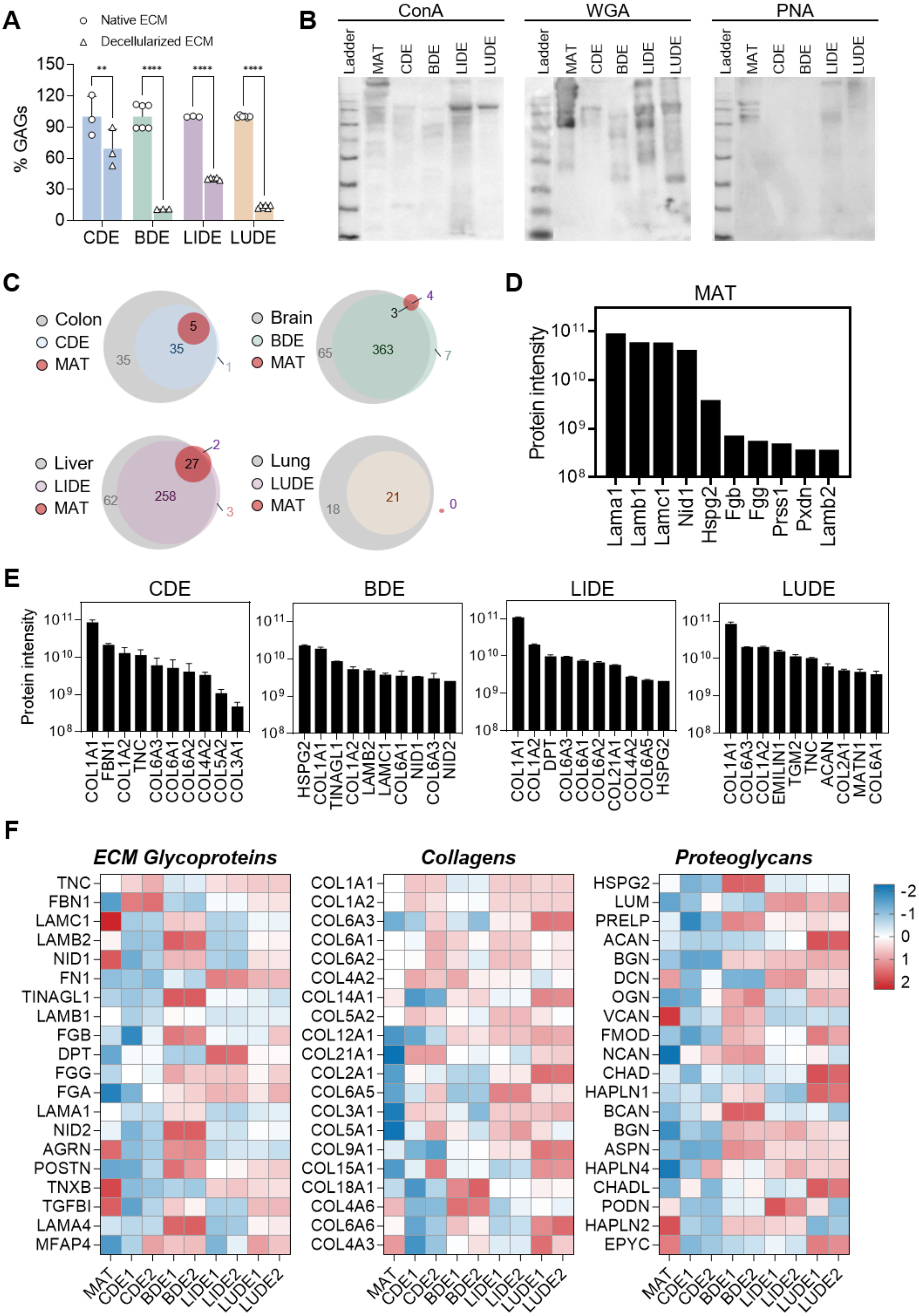

Supplementary Figure 1. Biochemical and proteomic characterization of tissue-specific dECMs. (A) Quantification of GAG content in native tissues and the corresponding dECM. (B)

Lectin blot analysis of MAT and tissue-derived dECM (CDE, BDE, LIDE, and LUDE) using concanavalin A (ConA), wheat germ agglutinin (WGA), and peanut agglutinin (PNA). (C) Venn diagram showing the overlap between proteins identified in each dECM and tissue-specific enriched proteins from the Human Protein Atlas (HPA). Top 10 most abundant matrisome proteins in (D) MAT and (E) tissue-derived dECM (CDE, BDE, LIDE, and LUDE). (F) Heatmaps showing the z-score-normalized abundance of the top 20 detected proteins in the glycoprotein, collagen, and proteoglycan subcategories of the matrisome across all tissue-derived dECMs.

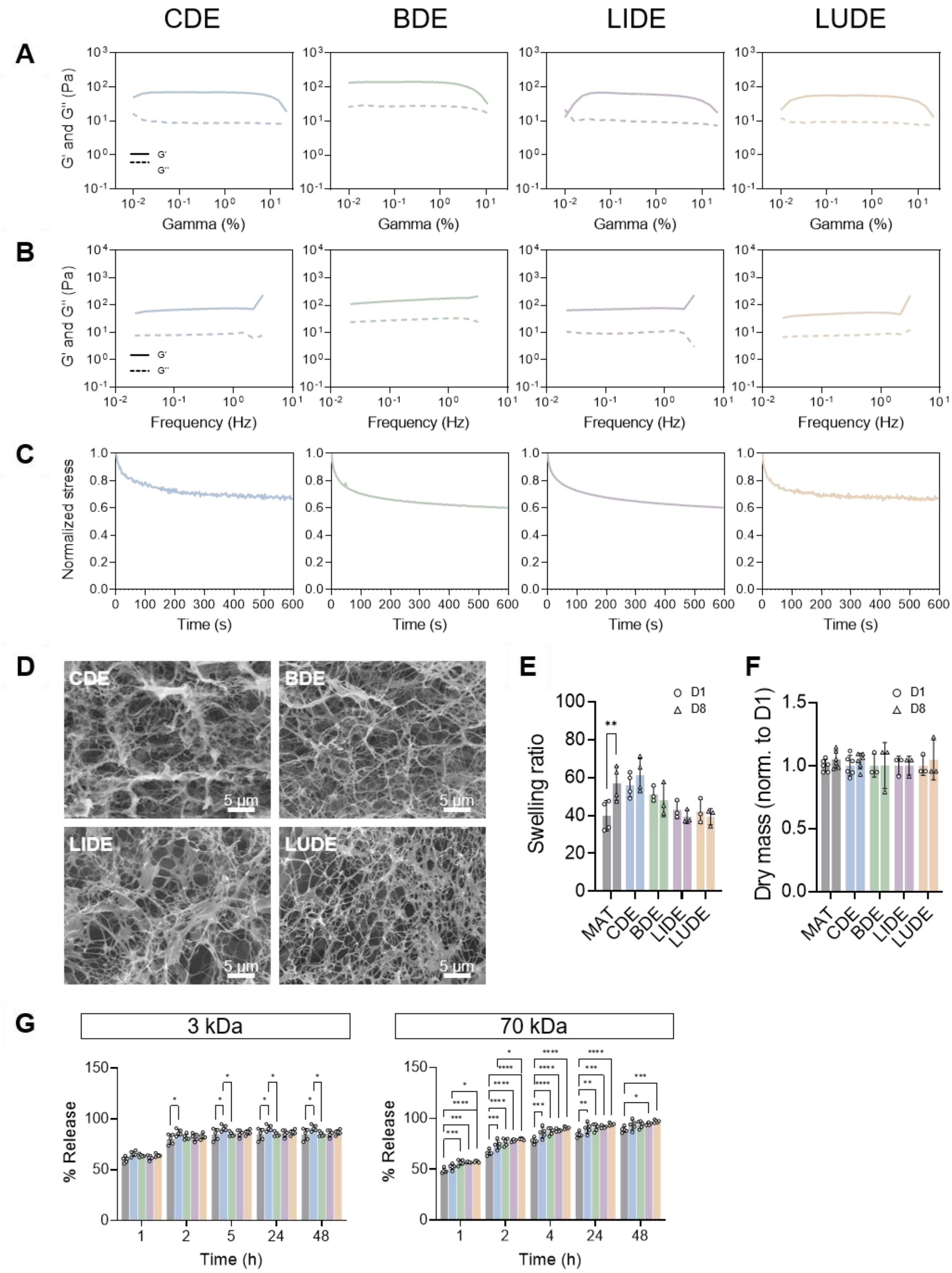

Supplementary Figure 2. Physical characterization of tissue-specific dECM hydrogels. (A, B) Strain sweep (A) and frequency sweep (B) analyses of storage (G′) and loss (G″) moduli for CDE, BDE, LIDE, and LUDE hydrogels. (C) Stress relaxation profiles of the dECM hydrogels. (D) Representative SEM images showing the microstructures of the dECM hydrogels. (E) Swelling ratio and (F) dry mass of the dECM hydrogels. (G) Cumulative dextran release (%) from the dECM hydrogels.

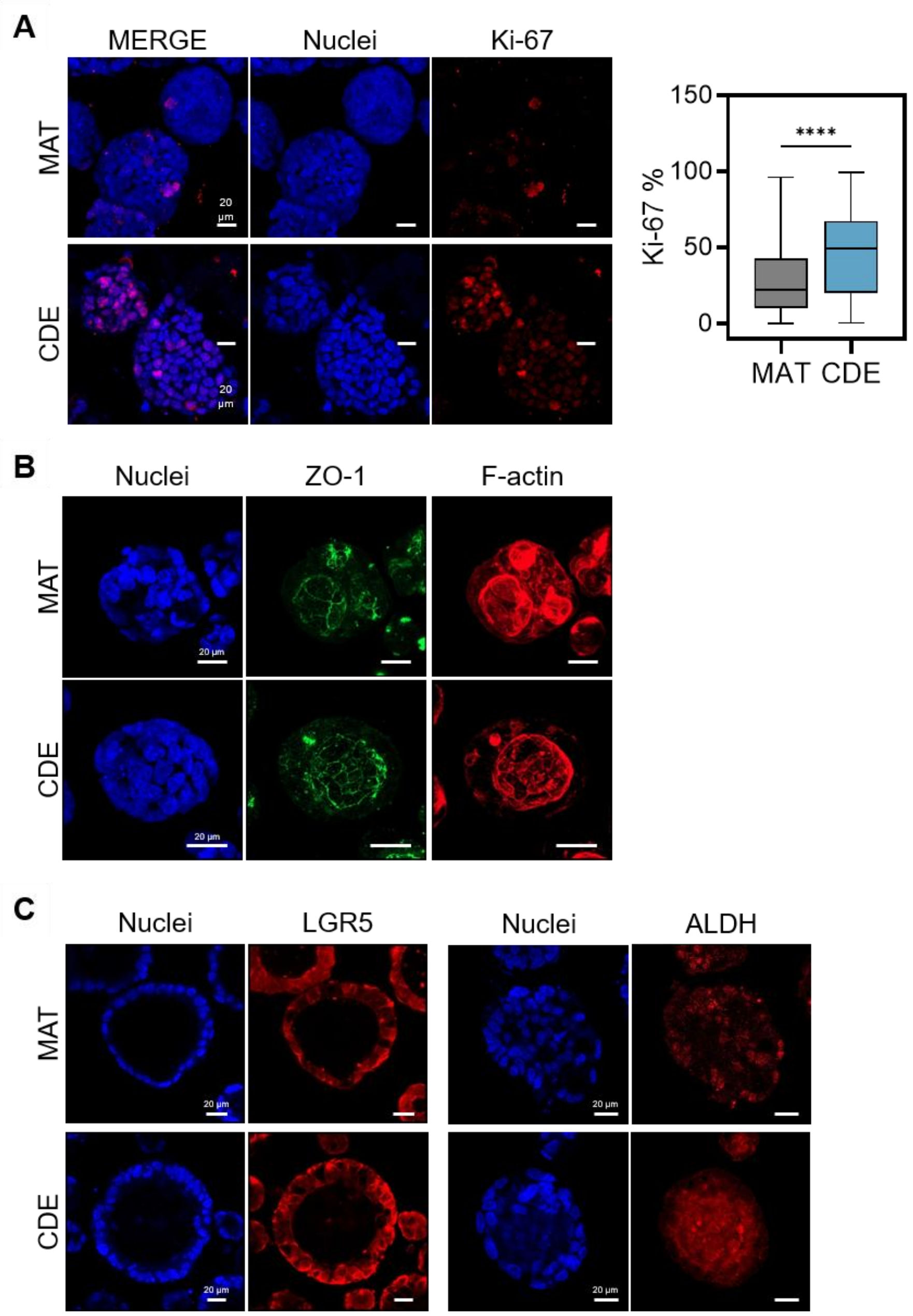

Supplementary Figure 3. Immunofluorescence characterization of PDCOs cultured in MAT and CDE. (A) Representative immunofluorescence images of Ki-67 staining (left) and quantification of Ki-67–positive cells (right). (B) Representative immunofluorescence images of the epithelial tight junction marker ZO-1 (green) and F-actin (red). (C) Representative immunofluorescence images of the CRC stem-like cell markers LGR5 and ALDH1. (A, B) Images were obtained as maximum intensity projections (MIP).

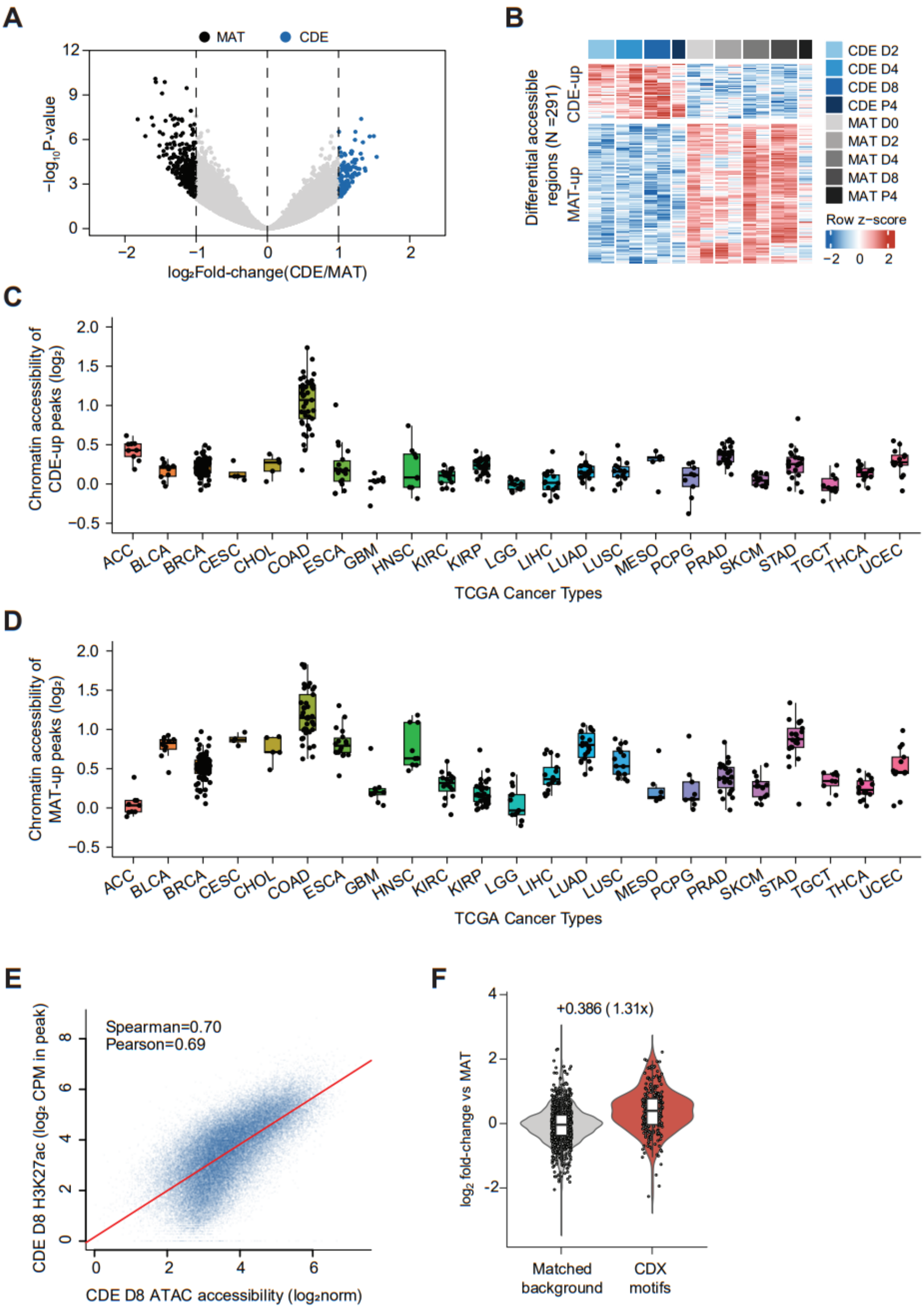

Supplementary Figure 4. CDE-induced chromatin accessibility changes align with human CRC epigenetic signatures.

(A) Volcano plot of differential chromatin accessibility between PDCOs cultured in CDE and MAT (DESeq2). Black and blue dots denote regions with significantly higher accessibility in MAT and CDE, respectively (dashed lines, $|\log_2$ fold-change$| = 1$). (B) Heatmap of the 291 differentially accessible regions from (A), grouped as CDE-up and MAT-up, across CDE and MAT cultures at the indicated time points (P4, Passaged 4 times). Signal is scaled by row z-score. (C, D) Boxplots of average chromatin accessibility of (C) CDE-up and (D) MAT-up peaks across 23 cancer types in TCGA [1]. Each dot represents an individual patient sample. (E)

Scatter plot of H3K27ac CUT&Tag signal ($\log_2$ CPM in peaks) versus chromatin accessibility (normalized and log2 transformed read counts) in PDCOs cultured in CDE at day 8. Each point represents one region of the ATAC-seq consensus peak set (n=73,118). Spearman's ρ and Pearson's r are indicated; the red line is linear regression fit. (F) Violin plots of H3K27ac $\log_2$ fold-change (CDE versus MAT, day 8) at CDX motif–containing accessible regions (n=268) and peak-strength–matched background regions. CDX motif regions showed a mean increase of 0.386 in $\log_2$ scale (1.31-fold; Wilcoxon rank-sum $P = 5 \times 10^{-8}$; permutation $P < 10^{-5}$). Embedded boxes indicate the median and interquartile range; whiskers extend to 1.5 × the interquartile range from the box hinges. Two biological replicates per condition.

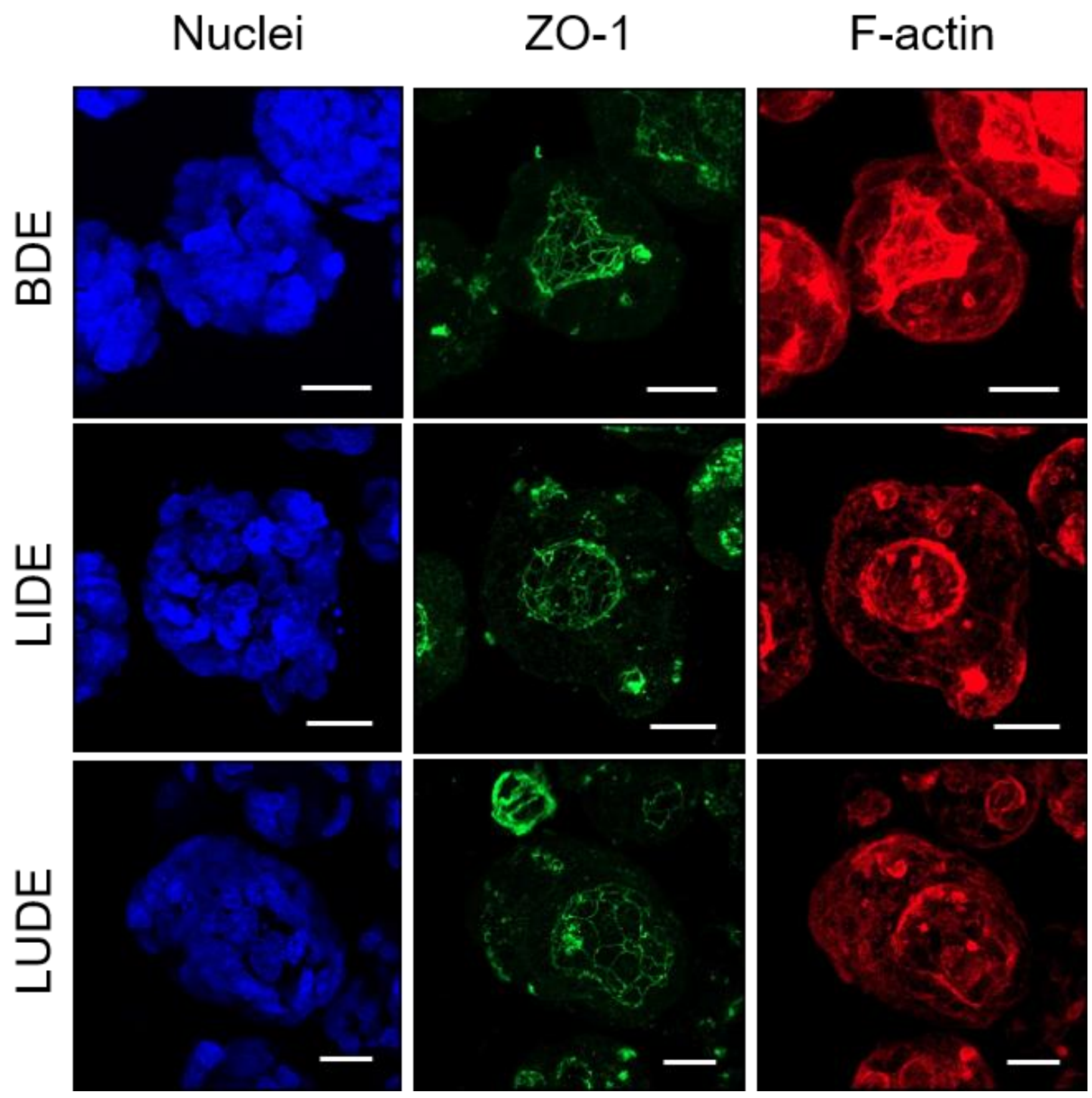

Supplementary Figure 5. Immunofluorescence analysis of ZO-1 expression in PDCOs. Representative immunofluorescence images of the epithelial tight junction marker ZO-1 (green) and F-actin (red). Images were obtained as maximum intensity projections (MIP).

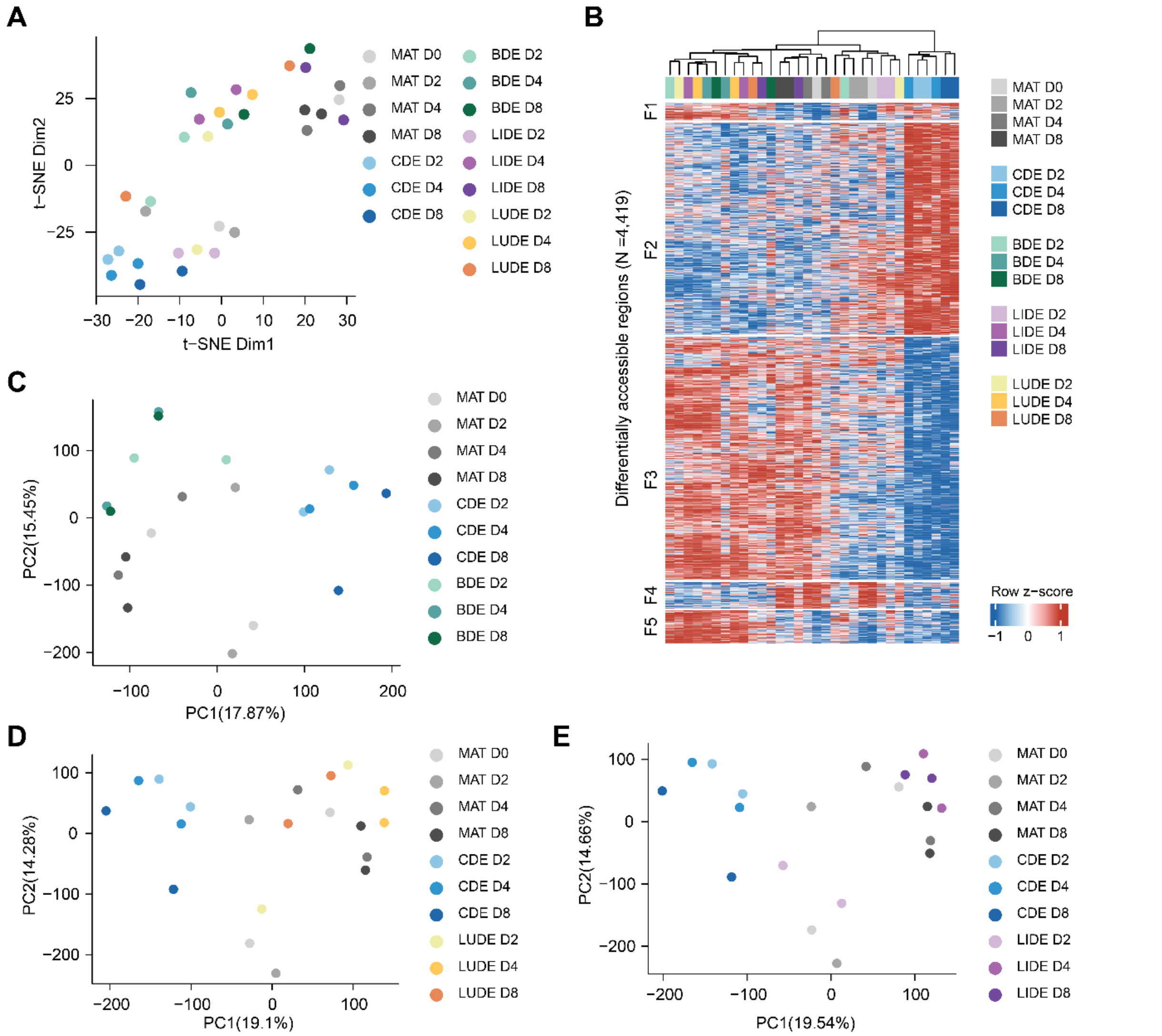

Supplementary Figure 6. Chromatin accessibility landscape across tissue-specific ECMs.

(A) A t-Distributed Stochastic Neighbor Embedding (t-SNE) analysis with perplexity 6 of PDCOs cultured in MAT, CDE, BDE, LIDE, and LUDE harvested at days 0, 2, 4, and 8. (B) Heatmap of differentially accessible chromatin peaks across culture conditions. Hierarchical clustering reveals striking differences between CDE and other culture conditions. Peaks are categorized into five distinct features: F1 (accessible in all conditions except MAT), F2 (exclusively accessible in CDE), F3 (accessible in all conditions except CDE), F4 (exclusively accessible in MAT), and F5 (accessible in BDE, LIDE, and LUDE). (C-E) Principal component analysis providing detailed visualization of chromatin accessibility distribution in (C) BDE, (D) LIDE, and (E) LUDE conditions, depicting direct comparison with MAT and CDE reference conditions.

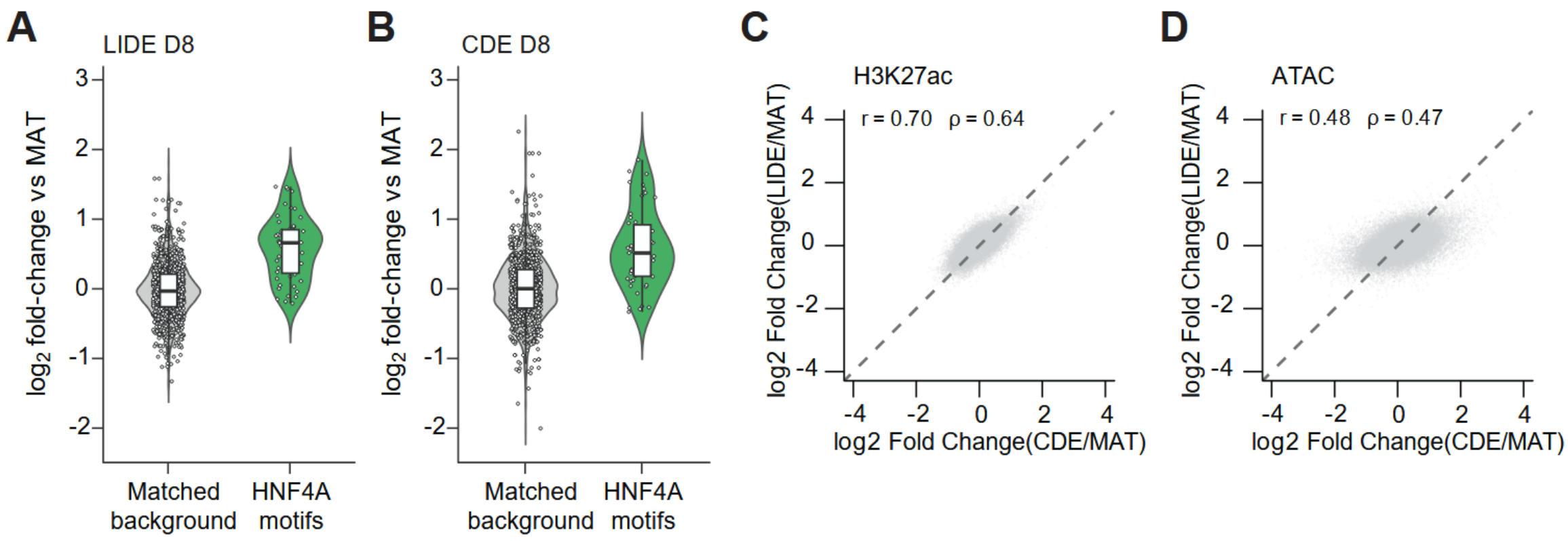

Supplementary Figure 7. H3K27ac gain at lineage-associated regions is shared across tissue-derived dECMs, whereas chromatin accessibility changes are tissue-specific.

(A, B) Violin plots of H3K27ac CUT&Tag $\log_2$ fold-change relative to MAT at day 8 for HNF4A motif–containing accessible regions (n=51) and peak-strength–matched background regions, in PDCOs cultured in (A) LIDE and (B) CDE. The same LIDE-defined region set was analyzed in both panels. HNF4A motif regions gained H3K27ac relative to matched background in LIDE (1.57-fold; permutation $P < 10^{-5}$) and also in CDE (1.42-fold; permutation $P < 10^{-5}$), despite HNF4A being non-cognate to the colon-derived matrix. Embedded boxes indicate the median and interquartile range; whiskers extend to the most extreme value within 1.5 × the interquartile range of the hinges. Two biological replicates per condition. (C, D) Scatter plots comparing $\log_2$ fold-changes relative to MAT between LIDE and CDE at day 8 for (C) H3K27ac and (D) chromatin accessibility (ATAC). Pearson's r and Spearman's ρ are indicated; dashed line is identity line (y = x).

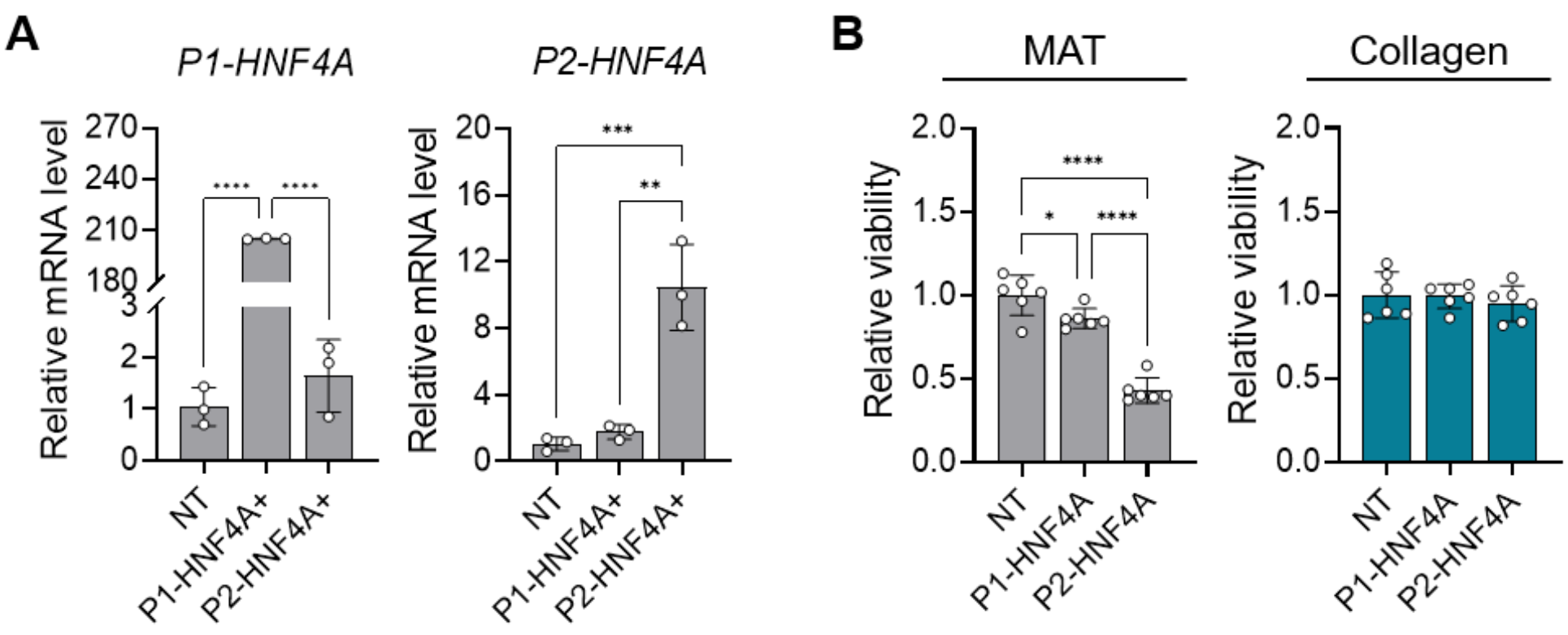

Supplementary Figure 8. Validation and functional assessment of HNF4A overexpression in PDCOs.

(A) Quantitative PCR validation of P1- and P2-HNF4A overexpression in PDCOs following lentiviral transduction. Relative expression levels were normalized to NT controls. (B) Viability of NT control, P1-HNF4A-overexpressing, or P2-HNF4A-overexpressing PDCOs cultured in MAT or collagen type I hydrogels at a concentration matched to that used for LIDE culture, as assessed by the WST-8 assay. Data are presented as mean ± SD ($n \geq 3$). Statistical significance was determined using one-way ANOVA with Tukey's multiple comparisons test. ns, not significant; *$P < 0.05$, **$P < 0.01$, ***$P < 0.001$, and ****$P < 0.0001$.

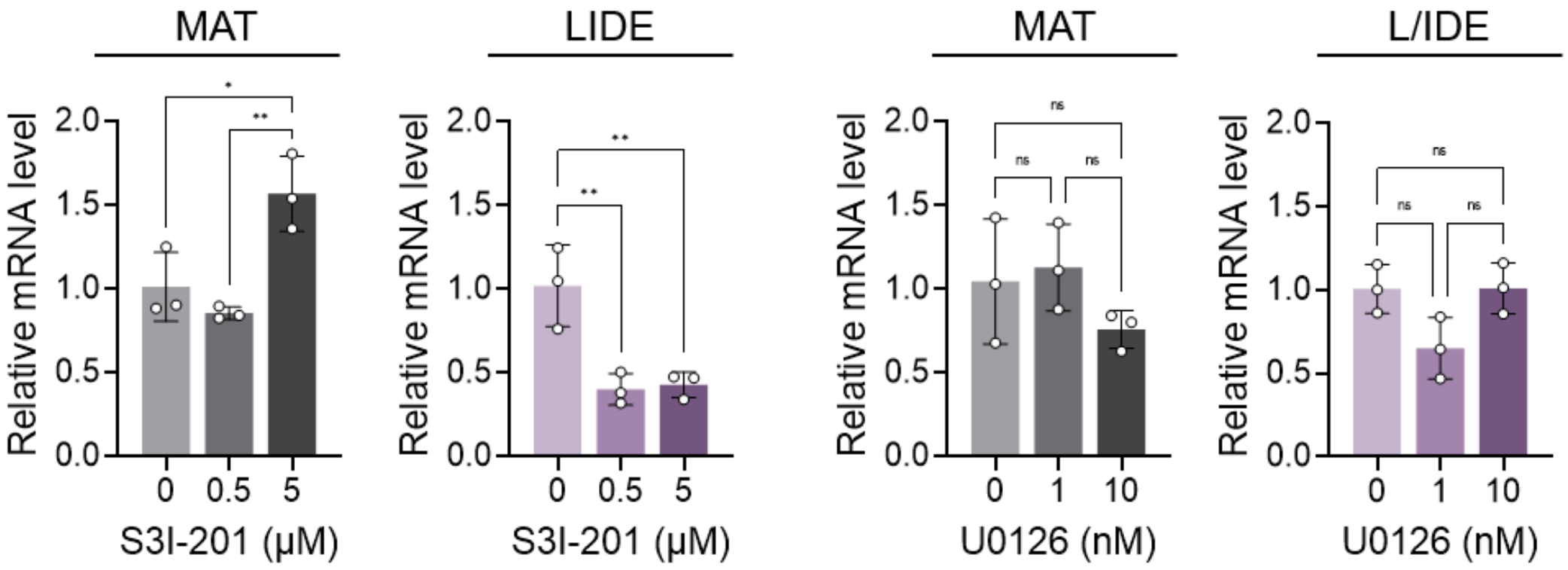

Supplementary Figure 9. Assessment of signaling pathways associated with P2-HNF4A expression in PDCOs. PDCOs cultured in MAT or LIDE hydrogels were treated with the STAT3 inhibitor S3I-201 or the ERK inhibitor U0126 for 8 days, and P2-HNF4A expression was evaluated by qPCR. Data are presented as mean ± SD (n ≥ 3). Statistical significance was determined using one-way ANOVA with Tukey's multiple comparisons test. ns, not significant; *P < 0.05, **P < 0.01, ***P < 0.001, and ****P < 0.0001.

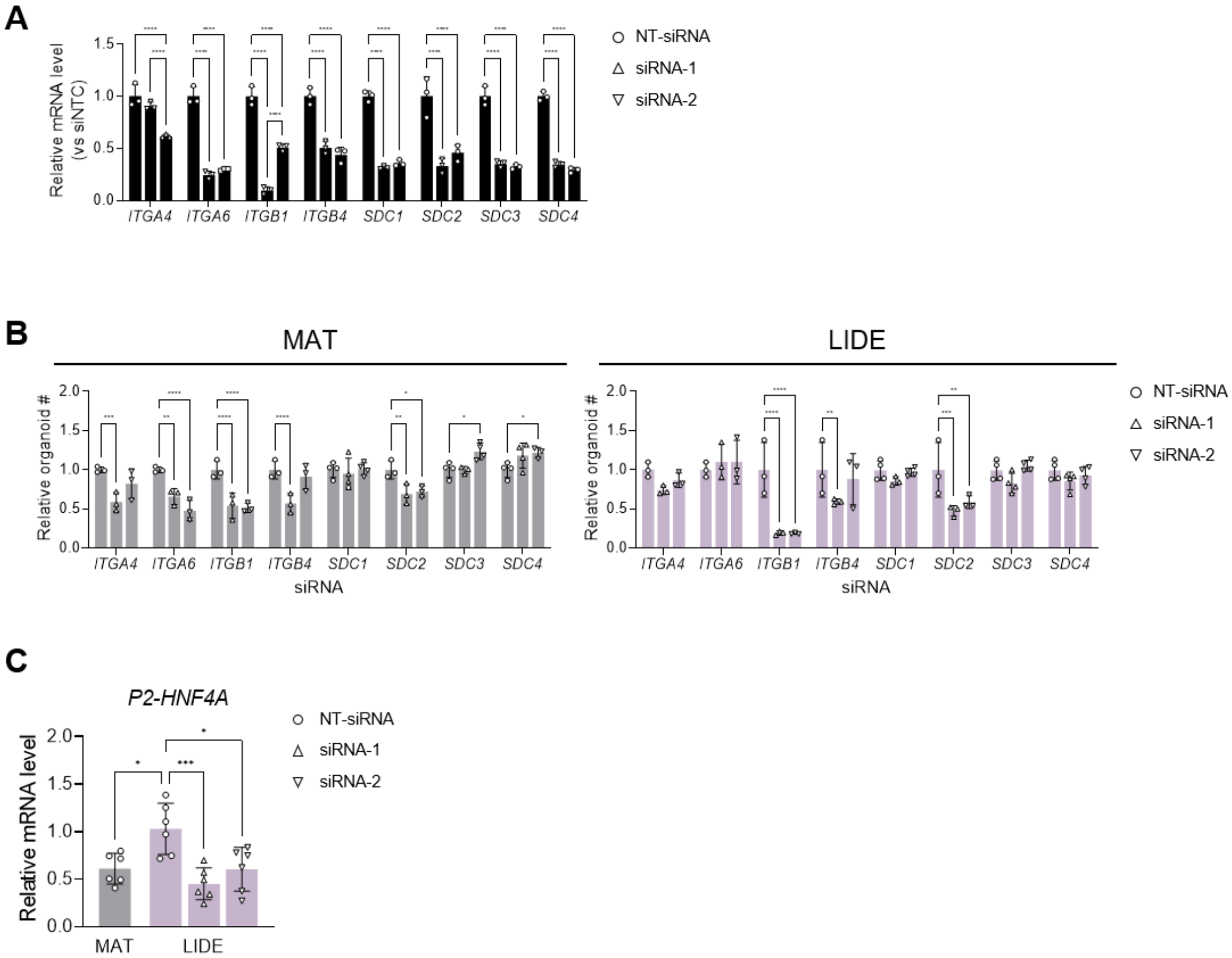

Supplementary Figure 10. Integrin-mediated regulation of organoid formation and P2-HNF4A expression in PDCOs cultured in MAT or LIDE.

(A) Validation of siRNA-mediated knockdown efficiency of the indicated integrin (*ITGA4, ITGA6, ITGB1,* and *ITGB4*) and syndecan (*SDC1, SDC2, SDC3,* and *SDC4*) genes in PDCOs by qPCR. (B) Screening of the effects of knockdown of the indicated integrin and syndecan genes on organoid formation in PDCOs cultured in MAT or LIDE for 8 days. (C) Relative P2-HNF4A mRNA expression in ITGB1 siRNA-transfected PDCOs cultured in LIDE, as determined by quantitative PCR. Organoid formation was assessed on day 8 after culture. Data are presented as mean ± SD ($n \geq 3$). Statistical significance was determined using one-way ANOVA with Tukey's multiple comparisons test. *$P < 0.05$, **$P < 0.01$, ***$P < 0.001$, and ****$P < 0.0001$.

Supplementary materials and methods

A. Glycosaminoglycan (GAG) Content Analysis

The glycosaminoglycan (GAG) content of each dECM was quantified using a dimethylmethylene blue (DMMB) assay kit (Blyscan, USA) according to the manufacturer's instructions.

B. Lectin blot analysis

Lyophilized dECM samples were lysed in RIPA buffer containing a protease inhibitor cocktail, followed by sonication to facilitate protein extraction. After centrifugation, the collected supernatants were prepared with 4× sample buffer and subjected to SDS-PAGE. Separated proteins were transferred onto PVDF membranes for lectin blot analysis. The membranes were blocked with 3% bovine serum albumin (BSA) in TBST and incubated overnight at 4°C with biotinylated Concanavalin A (ConA; Sigma-Aldrich, C2272) or Wheat Germ Agglutinin (WGA; Sigma-Aldrich, L5142). Following washing, the membranes were incubated with HRP-conjugated streptavidin (Thermo Fisher Scientific, N100). For detection of PNA-binding glycoconjugates, membranes were incubated overnight at 4 °C with peroxidase-labeled Peanut Agglutinin (PNA; Sigma-Aldrich, L7759). Lectin-reactive proteins were visualized using Pierce™ ECL Western Blotting Substrate (Thermo Fisher Scientific) and imaged using a ChemiDoc imaging system (Bio-Rad). Image acquisition and analysis were performed using Image Lab software (Bio-Rad).

C. Stress Relaxation Test

Stress relaxation measurements were performed using a HAAKE MARS III rheometer (Thermo Scientific, USA). Each dECM hydrogel sample was placed between the rheometer plates and equilibrated at 25 °C prior to measurement. A constant strain of 10% was applied, and the decrease in stress over time was monitored for 600 s to evaluate the stress relaxation behavior of each dECM hydrogel.

D. Scanning Electron Microscopy (SEM)

SEM imaging was performed to evaluate the microstructure of each dECM hydrogel after

gelation. The hydrogels were frozen, lyophilized, and coated with platinum using an Ion Sputter Coater (SPT-20, Coxem, Republic of Korea). The surface morphology of the hydrogels was observed using a scanning electron microscope (Hitachi Model S-4800, Japan).

E. Swelling Ratio

The swelling properties of each dECM hydrogel were determined by measuring the wet weight at each time point, followed by freezing and lyophilization to obtain the corresponding dry weight. The swelling ratio was calculated using the following equation:

$$\text{Swelling ratio} = \frac{\mathrm{W}_{wet} - W_{dry}}{W_{dry}}$$

where W_{wet} is the wet weight of the hydrogel at each time point and W_{dry} is the corresponding dry weight after lyophilization. Three samples from each group (n = 3) were analyzed.

F. Dry Mass

The relative dry mass of each dECM hydrogel was evaluated under culture conditions. Following incubation in culture medium at 37 °C, the hydrogels were lyophilized and weighed at the indicated time points. The dry mass was normalized to the day 1 value of each dECM hydrogel.

G. Dextran release assay

Dextran release from dECM hydrogels was evaluated using fluorescently labeled dextran with different molecular weights, including fluorescein-conjugated dextran (70,000 MW; Thermo Fisher Scientific, D1823) and tetramethylrhodamine-conjugated dextran (3,000 MW; Thermo Fisher Scientific, D3307). Dextran-loaded dECM hydrogels were incubated in release medium under controlled conditions. At predetermined time points (1, 2, 4, 24, and 48 h), the released dextran was collected and quantified by fluorescence measurement. The cumulative release amount was calculated over time.

H. Lentiviral overexpression of HNF4A isoforms in PDCOs

Coding sequences for the P1- and P2-driven *HNF4A* isoforms (CCDS46605.1 and

CCDS46604.1, respectively) with an upstream CMV promoter were synthesized as gBlocks (IDT) and inserted by Gibson assembly (NEB #E2621) into a lentiviral vector maintained in our laboratory (modified from pU6-sgGFP-NT1; Addgene #46914). The vector's U6–sgRNA cassette was removed. The resulting constructs therefore express *HNF4A*, puromycin N-acetyltransferase, and mCherry from a single transcript (CMV–HNF4A–P2A–PuroR–T2A–mCherry), such that puromycin selection and mCherry fluorescence both report HNF4A expression. All plasmids were verified by Sanger sequencing.

Lentivirus was produced in HEK293T cells seeded on a 6-well plate. At 80% confluence, cells were co-transfected with lentiviral vector, psPAX2 (Addgene #12260), and pMD2.G (Addgene #12259) using Lipofectamine 3000 (Invitrogen, #L3000015) according to the manufacturer's instructions. Viral supernatant was collected at 72 h post-transfection, and concentrated by Lenti-X™ Concentrator (TaKaRa, #631232).

PDCOs were transduced with lentiviruses encoding NT, P1-*HNF4A*, or P2-*HNF4A* using a modified method based on a previously described Matrigel bilayer culture approach for organoid propagation and infection [1]. Following lentiviral transduction, organoids were treated with puromycin for selection. The selected organoids were subsequently expanded in Matrigel and used for further experiments.

I. Pharmacological inhibition of STAT3 and MEK/ERK signaling

To evaluate the potential involvement of STAT3- and ERK-associated signaling in P2-HNF4A regulation, PDCOs cultured in MAT or LIDE hydrogels were treated with the STAT3 inhibitor S3I-201 (0.5 and 5 μM) or the MEK/ERK inhibitor U0126 (1 and 10 nM) for 8 days. Following treatment, total RNA was extracted, and P2-*HNF4A* expression was analyzed by quantitative PCR as described above.

References

[1] Y. Maru, K. Onuma, M. Ochiai, T. Imai, Y. Hippo, Shortcuts to intestinal carcinogenesis by genetic engineering in organoids, Cancer Sci. 110(3) (2019) 858-866.
